# METTL3 promotes oral squamous cell carcinoma by regulating miR-146a-5p/SMAD4 axis

**DOI:** 10.18632/oncotarget.28717

**Published:** 2025-05-08

**Authors:** Jayasree Peroth Jayaprakash, Pragati Karemore, Piyush Khandelia

**Affiliations:** ^1^Laboratory of Molecular Medicine, Department of Biological Sciences, Birla Institute of Technology and Science, Pilani - Hyderabad Campus, Hyderabad 500078, India

**Keywords:** oral cancer, m6A RNA methylation, METTL3, miR-146a-5p, SMAD4

## Abstract

N6-methyladenosine (m6A), one of the most prominent and reversible internal modifications of eukaryotic RNAs, has emerged as a critical regulator of gene expression in various cancers including oral squamous cell carcinoma (OSCC), wherein it shapes the tumor-specific epitranscriptomic gene-regulatory networks. METTL3, the primary m6A RNA methyltransferase, is significantly upregulated in OSCC cells leading to increased global m6A levels. Interestingly, METTL3 positively regulates miRNA biogenesis by modulating the processing of primary miRNAs in a m6A-dependent manner. We identified miR-146a-5p, an oncogenic miRNA as one of the METTL3-regulated miRNAs in OSCC. METTL3-depletion or inhibition of its catalytic activity leads to a reduction of miR-146a-5p and an appreciable accumulation of primary miR-146a in OSCC cells. Functional assays examining the effects of miR-146a-5p inhibition or overexpression confirm its oncogenic role in OSCC pathophysiology. Further, SMAD4, a central transducer in TGF-β signaling, was identified as a miR-146a-5p target. In OSCC cells, SMAD4-depletion exacerbates the oncogenic traits, whereas its overexpression exerts the opposite effect. Additionally, METTL3-depletion dysregulates SMAD4-regulated genes suggesting its potential involvement in SMAD4-dependent TGF-β signaling. Taken together, we report that METTL3, an oncogene regulates the expression of SMAD4, a tumor-suppressor via miR-146a-5p, thus unveiling a novel regulatory axis of METTL3/miR-146a-5p/SMAD4 in OSCC, which can potentially have therapeutic implications.

## INTRODUCTION

Oral squamous cell carcinoma (OSCC), which develops in the oral mucosa, is one of the most common and aggressive forms of cancer in the head and neck region [1]. OSCC is a growing global health concern, accounting for 389,485 new cases and 188,230 deaths annually, and a five-year mortality rate of 50% that has remained largely unchanged in the past 40 years [2]. The primary treatment for OSCC involves surgical resection followed by chemotherapy and radiotherapy. However, a major challenge in treating OSCC is the difficulty in diagnosing it at an early stage due to the lack of specific signs and symptoms, which often leads to misdiagnosis or advanced-stage detection. Recurrence, tumor invasion, drug resistance, and both local and distant metastases are the leading causes of death in OSCC patients [3].

N6-methyladenosine (m6A) RNA methylation, a well-studied reversible epitranscriptomic modification of RNA, fine-tunes RNA structure and function, thus influencing gene expression by impacting various facets of RNA metabolism such as splicing, stability, nuclear export, and translation, in both normal development and diseased scenarios [4, 5]. Dysregulation of various m6A components i.e., m6A methyltransferases, m6A demethyltransferases, and m6A binding proteins, is frequently associated with the progression of multiple cancers, primarily via regulating cell growth, metastasis, and cell death [6]. Expression of the principal m6A methyltransferase i.e., methyltransferase-like 3 (METTL3) and global m6A levels are elevated in OSCC [7–9]. METTL3 promotes proliferation, resistance to apoptosis, invasion, lymph node metastasis, epithelial-mesenchymal transition (EMT), chemo-radio resistance, and stem cell properties in OSCC and leads to poor prognosis and shorter survival of OSCC patients [10–15]. In addition, STM2457, a small-molecule inhibitor of METTL3’s catalytic activity, confers drug sensitivity to OSCC cells both *in vitro* and *in vivo* [16]. METTL3 regulates the biogenesis of a subset of microRNAs (miRNAs) in an m6A RNA methylation-dependent manner requiring its catalytic activity, wherein the recognition of m6A marked primary miRNAs (pri-miRNAs) by the microprocessor complex subunit, DiGeorge syndrome critical region 8 (DGCR8) is favored, thus enhancing the maturation of pri-miRNAs to miRNAs [17]. miRNAs are short, non-coding regulatory RNAs reported to be differentially expressed in several cancers, including OSCC [18–20]. Several METTL3/m6A-regulated miRNAs have since been characterized in various cancers, and have been shown to regulate various aspects of cancer pathology [21]. METTL3/m6A-regulated miRNAs such as miR-99a-5p, miR-31-5p, and miR-151-5p have been implicated in OSCC progression [22–24]. SMAD4 is a pivotal signal transducer in the canonical Transforming Growth Factor-β (TGF-β) pathway, which forms a heterotrimeric complex with SMAD2/3 and translocates to the nucleus to suppress tumor progression [25]. However, in later stages of cancer, SMAD4’s genetic/epigenetic alterations impair TGF-β’s tumor-suppressive function [25], wherein the expression of transcription factors such as snail family transcriptional repressor 1 (SNAIL), snail family transcriptional repressor 2 (SLUG), zinc finger E-box binding homeobox 1 (ZEB1) and zinc finger E-box binding homeobox 2 (ZEB2) is enhanced leading to upregulation of mesenchymal markers (N-cadherin, vimentin, fibronectin) and downregulation of epithelial markers (E-cadherin, Claudin-1) [26]. SMAD4 expression is lower in OSCC tissue samples than in normal oral mucosa and in OSCC cell lines compared to normal oral keratinocytes [27].

In the present study, we uncover the regulation of miR-146a-5p by METTL3 in OSCC cells, a miRNA frequently overexpressed in OSCC patients [28–30]. Our data ascribe an oncogenic role to METTL3 in OSCC progression. We show that depletion of METTL3 or treatment with its catalytic inhibitor i.e., STM2457, leads to the accumulation of primary miR-146a in OSCC cells with a concomitant decrease in the mature miR-146a-5p levels. In addition, the phenotype observed upon METTL3-depletion in OSCC cells is rescued by overexpression of miR-146a-5p. Furthermore, our results indicate that the METTL3/miR-146a-5p mediated oncogenic effect in OSCC is via direct targeting of SMAD4, a well-known tumor-suppressor gene, by miR-146a-5p. Further, we show a tumor-suppressive role for SMAD4 in OSCC cells. Interestingly, METTL3-depletion reduces the expression of downstream effectors of SMAD4 such as N-cadherin, SNAIL, SLUG, and ZEB1, suggestive of its involvement in the regulation of SMAD4-dependent TGF-β signaling in OSCC cells. Taken together, this study not only conclusively shows the novel regulatory role of METTL3/miR-146a-5p/SMAD4 axis in OSCC progression, but also makes it a potential therapeutic target.

## RESULTS

### METTL3 depletion suppresses OSCC progression by regulating cell proliferation, apoptosis, migration, and invasion

**Figure 1:**
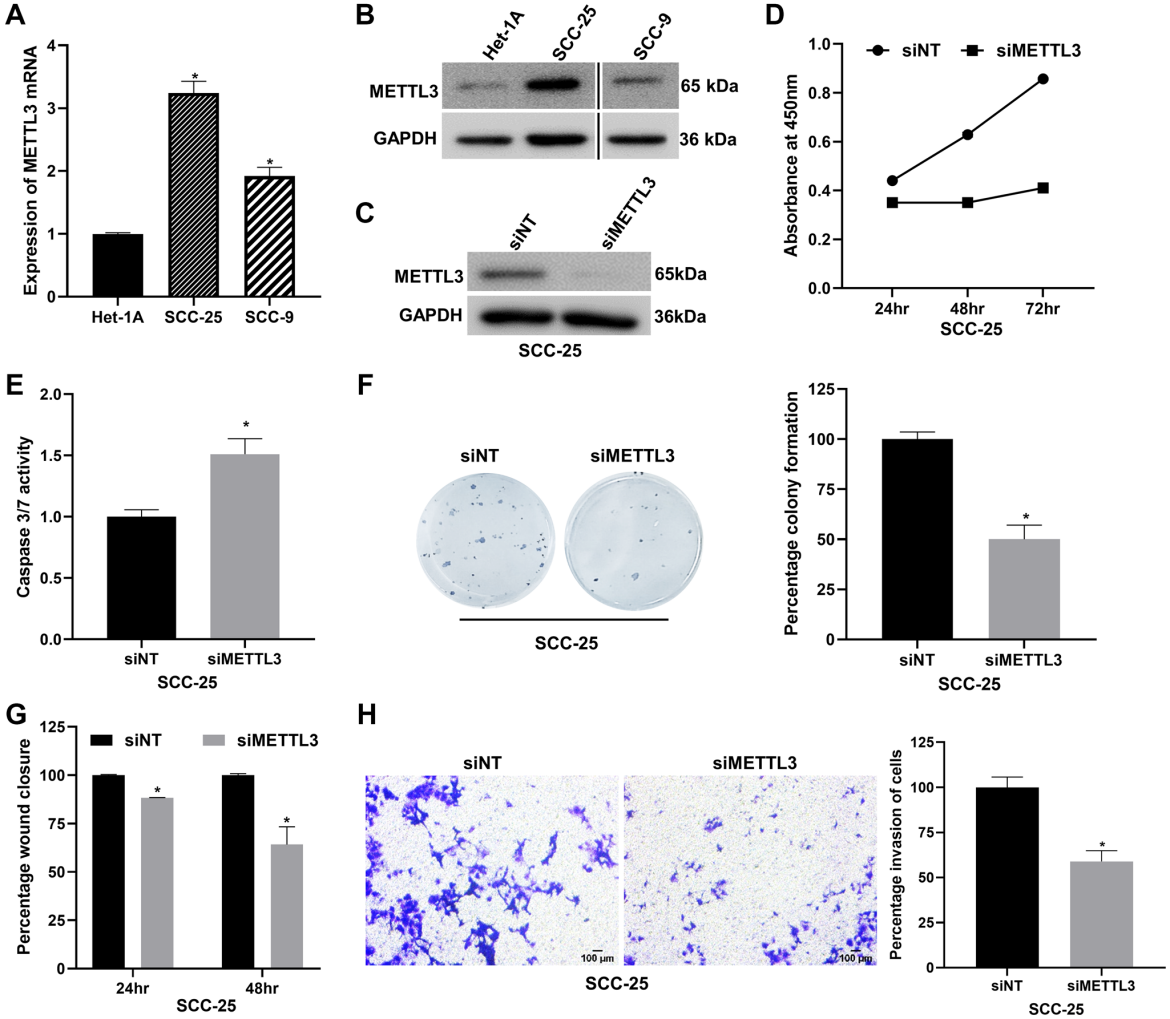
METTL3 depletion reduces proliferation, migration, invasion and promotes apoptosis of the SCC-25 OSCC cell line *in vitro*. (**A**) RT-qPCR to examine the expression of METTL3 mRNA in OSCC cell lines SCC-25 and SCC-9 compared to normal oesophageal epithelial cell line Het-1A. (**B**) Western blot analysis showing the expression of METTL3 protein in SCC-25 and SCC-9 cell lines compared to normal cell line Het-1A. (**C**) Validation of siRNA-mediated knockdown of METTL3 in SCC-25 cells by western blot assay. (**D**) WST-1 assay showing the effect of METTL3 depletion on cell viability of SCC-25 cells at various time points. (**E**) Apoptosis assay in METTL3-depleted SCC-25 cells using caspase 3/7 luminometric assay. (**F**) Colony formation assay to determine the effect of METTL3 silencing on SCC-25 cells compared to control cells. (**G**) Wound healing assay to demonstrate the impact of METTL3 knockdown on the migration of SCC-25 at various time points. (**H**) Matrigel invasion assay showing the effect of METTL3 knockdown on SCC-25 cell invasion. Statistical comparisons were made using the student’s *t*-test and the data points represent the mean ± SEM. *P* < 0.05 was considered significant and the asterisk sign (^*^) denotes significant change compared to respective control samples.

Elevated expression of METTL3, the primary catalytic subunit of m6A RNA methylation, has been reported in OSCC [7, 8]. We verified the expression of METTL3 in two independent OSCC cell lines, SCC-25 and SCC-9 by RT-qPCR and western blot assays. We found that it is appreciably upregulated in both the cell lines compared to the normal oesophageal epithelial cell line Het-1A (Figure 1A, 1B and Supplementary Figure 1A). Next, to probe METTL3’s function in OSCC, we first established siRNA-mediated knockdown of METTL3 in SCC-25 and SCC-9 cells and achieved >90% reduction in METTL3 protein levels (Figure 1C and Supplementary Figure 1B). The WST-1 cell proliferation assay revealed a significant time-dependent reduction in SCC-25 cell viability, with ~44% and ~52% observed at 48 and 72 hours post-METTL3 depletion (Figure 1D). A similar time-dependent reduction in cell viability was observed in SCC-9 cells upon METTL3 knockdown (Supplementary Figure 1C). METTL3 is known to promote cell cycle progression in cancers like HNSCC and bladder cancer [31, 32] and it regulates cell cycle markers such as CDK2 (cyclin-dependent kinase 2) and Cyclin D1 in gastrointestinal stromal tumors [33]. To investigate METTL3’s role in OSCC proliferation at a molecular level, we measured cell cycle markers in METTL3-depleted SCC-25 cells via RT-qPCR. Knockdown of METTL3 reduced mRNA levels of positive regulators of the cell cycle such as CCNB1 (Cyclin B1) and CCND1 (Cyclin D1) (Supplementary Figure 1D), suggesting METTL3’s involvement in OSCC cell cycle progression. Furthermore, we observed a ~1.5-fold increase in caspase 3/7 activity in METTL3-depleted SCC-25 cells compared to control cells, indicative of METTL3’s role in regulating apoptosis of OSCC cells (Figure 1E). Akin to the cell viability, the colony formation ability of SCC-25 cells was also reduced by ~50% upon knockdown of METTL3 (Figure 1F). We further examined the role of METTL3 in cell migration and invasion of OSCC cells. The wound healing assay revealed that METTL3 silencing reduced the migration of SCC-25 cells by ~35% at 48 hours (Figure 1G and Supplementary Figure 1E). The matrigel invasion assay led to a ~40% and ~30% inhibition of cell invasion in SCC-25 and SCC-9 cells respectively upon METTL3 depletion (Figure 1H and Supplementary Figure 1F). Taken together, these results convincingly prove the oncogenic role of METTL3 in OSCC cell lines SCC-25 and SCC-9.

### METTL3 promotes OSCC progression by regulating miR-146a-5p maturation

**Figure 2:**
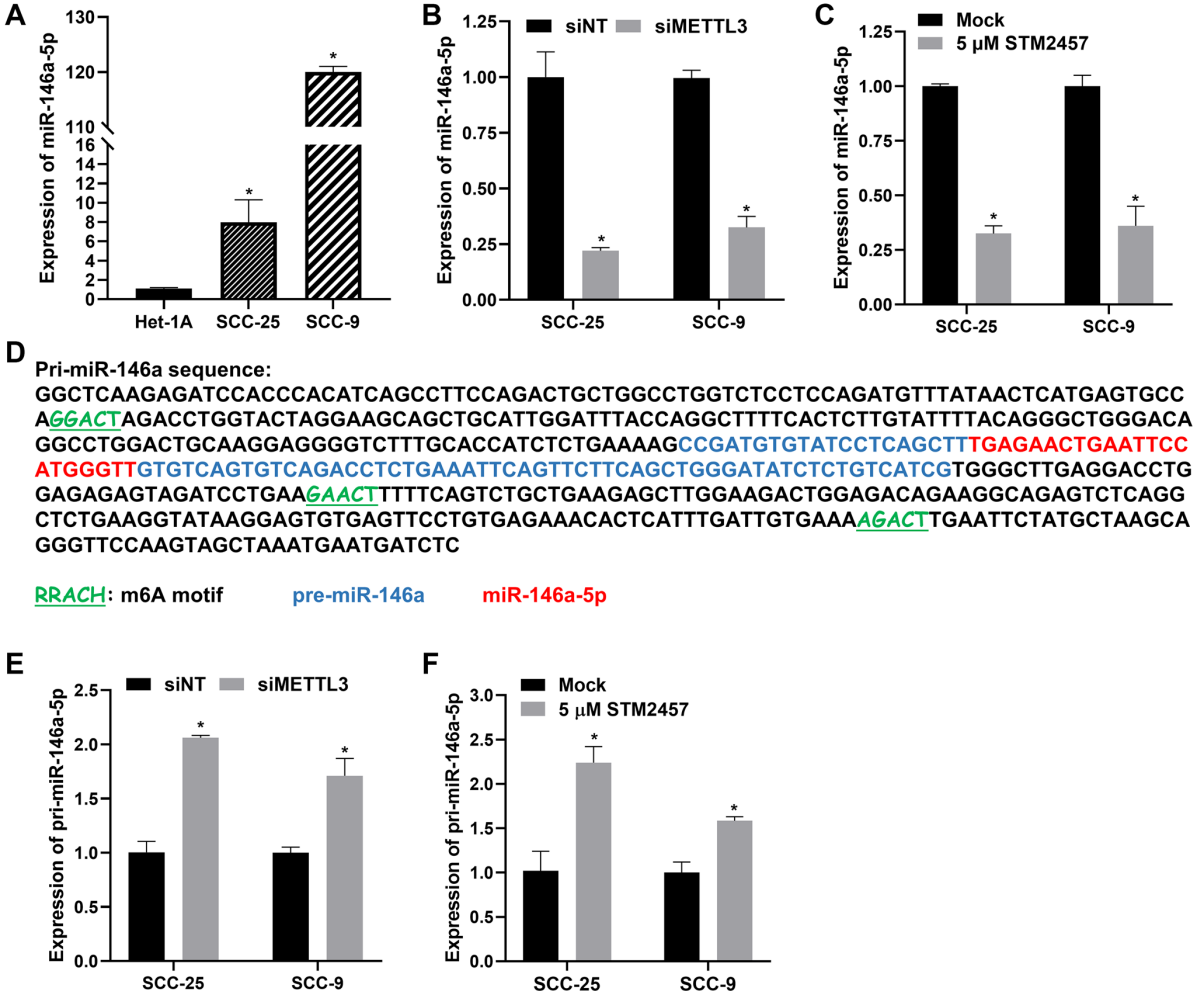
Regulation of miR-146a-5p expression by METTL3 in OSCC cells. (**A**) Quantification of mature miR-146a-5p expression in OSCC cell lines SCC-25 and SCC-9 compared to normal oesophageal epithelial cell line Het-1A by RT-qPCR assay. (**B**) Evaluation of mature miR-146a-5p expression in OSCC cell lines upon siRNA-mediated knockdown of METTL3 (**C**) Impact of STM2457 treatment on mature miR-146a-5p levels in OSCC cells, measured by RT-qPCR assay. (**D**) Pri-miR-146a sequence highlighting the potential m6A methylation motifs (underlined and labeled in green) predicted by the bioinformatics tool SRAMP. The pre-miR-146a and the mature miR-146a-5p sequences are represented in blue and red color respectively. (**E**) Impact of METTL3 knockdown on pri-miR-146a levels in SCC-25 and SCC-9 cells examined by RT-qPCR. (**F**) The relative level of pri-miR-146a in STM2457 treated OSCC cells measured by RT-qPCR. Statistical comparisons of the RT-qPCR data were made using the Student’s *t*-test and the data points represent the mean ± SEM. *P* < 0.05 was considered significant and the asterisk sign (^*^) denotes significant change compared to respective control samples.

METTL3 has been reported to exert its oncogenic role in bladder cancer by regulating the maturation of miR-146a-5p in an m6A-dependent manner, wherein METTL3 knockdown led to an accumulation of pri-miR-146a and thereby reduced the expression of mature miR-146a-5p [34]. miR-146a-5p is reported to be upregulated in OSCC patients, correlating with poor overall survival [28, 30, 35]. However, the regulatory link between miR-146a-5p and METTL3 in OSCC is unexplored and hence we hypothesized that METTL3 might drive OSCC progression via miR-146a-5p regulation. To begin with, we examined the expression pattern of mature miR-146a-5p in OSCC cell lines and found it to be upregulated by ~8 fold in SCC-25 and ~120 fold in SCC-9 compared to normal control Het-1A cells (Figure 2A). Next, to assess the role of METTL3 in regulating the levels of the mature miR-146a-5p, we measured mature miR-146a-5p levels in METTL3-depleted OSCC cells by RT-qPCR assay. Interestingly, we found an appreciable reduction in mature miR-146a-5p levels upon METTL3 knockdown in both the OSCC cell lines (Figure 2B). Next, we treated both the OSCC cells with STM2457, a small molecule inhibitor of the catalytic activity of METTL3 [16] and measured mature miR-146a-5p levels. Our data indicate a >2.5-fold decrease in miR-146a-5p levels upon STM2457 treatment, suggestive of a clear role of METTL3’s catalytic activity i.e. depositing m6A marks, in regulating miR-146a-5p levels in OSCC (Figure 2C).

A previous study by Alarcon et al., (2015) reported that METTL3-mediated m6A methylation on primary miRNA enhances its recognition by DGCR8, thereby promoting the processing and expression of mature miRNAs [17]. Based on this premise, we probed the presence of m6A sites on pri-miR-146a (499-nucleotide sequence obtained from UCSC Genome Browser), employing the computational tool ‘SRAMP’ [36], and identified three high-confidence ‘RRACH’ m6A motifs on pri-miR-146a (Figure 2D), with additional lower-confidence motifs and corresponding prediction scores detailed in Supplementary Figure 2A. Next, we analyzed the expression of pri-miR-146a in METTL3-depleted as well as STM2457-treated OSCC cells using RT-qPCR assay. The primers used for RT-qPCR assay are schematically represented in Supplementary Figure 2B. Interestingly, we found the accumulation of pri-miR-146a in OSCC cells upon METTL3 depletion (Figure 2E), as well as upon STM2457-mediated inhibition of METTL3’s catalytic activity (Figure 2F). The data conclusively show the requirement of METTL3’s catalytic activity i.e., m6A deposition, in promoting the maturation and thus levels of mature miR-146a-5p in OSCC cells.

### miR-146a-5p modulates OSCC progression *in vitro*

**Figure 3:**
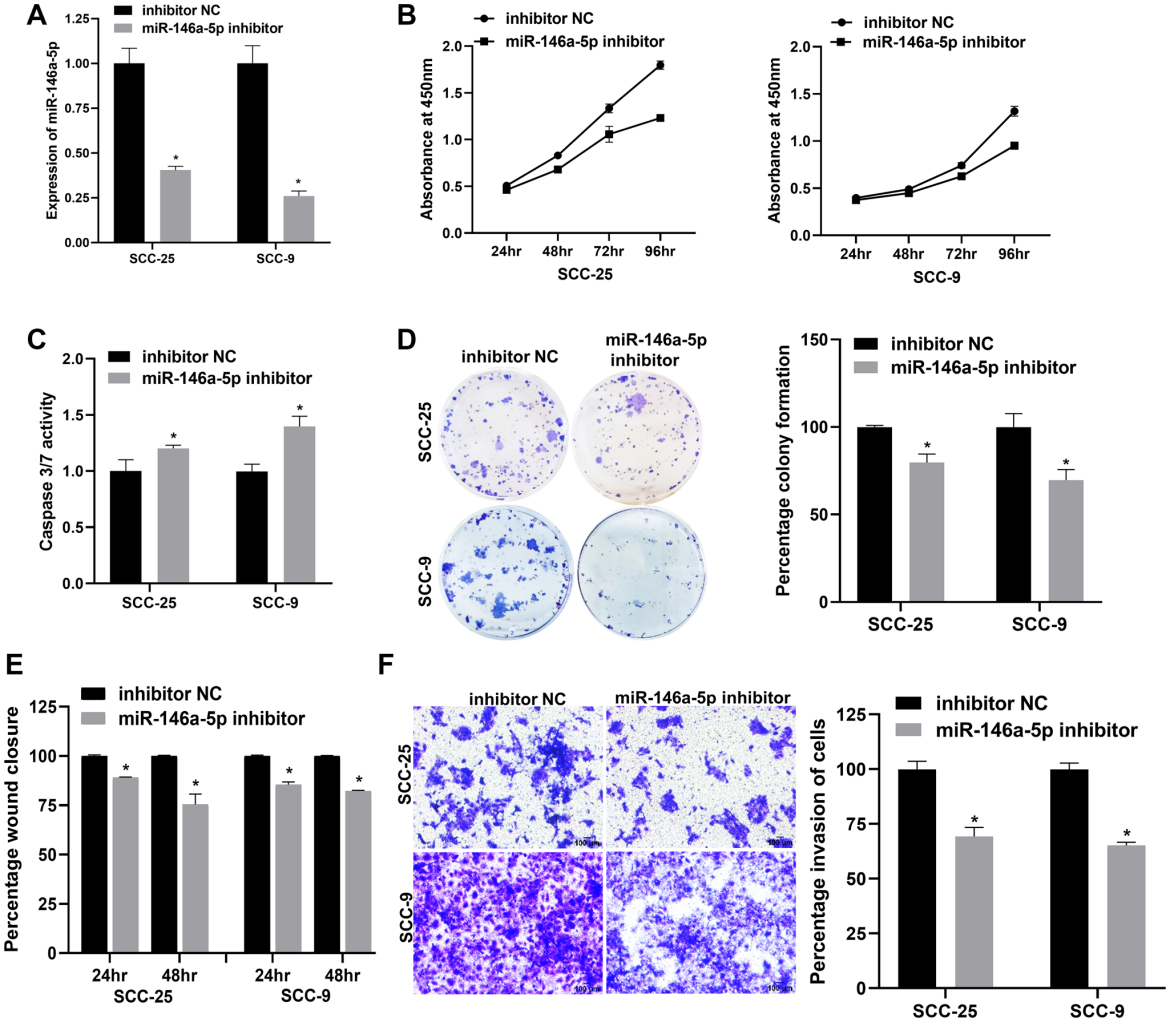
miR-146a-5p regulates the proliferation, apoptosis, migration, and invasion of OSCC cells *in vitro*. (**A**) Validation of miR-146a-5p knockdown in OSCC cell lines SCC-25 and SCC-9 by RT-qPCR. (**B**) WST-1 assay showing the effect of miR-146a-5p inhibition on the viability of SCC-25 and SCC-9 cells at various time points. (**C**) Activation of caspase 3/7 enzymes upon miR-146a-5p inhibition in OSCC cell lines measured by a luminometric caspase 3/7 assay. (**D**) Colony formation ability of SCC-25 and SCC-9 cells evaluated upon inhibition of miR-146a-5p. (**E**) Percentage wound closure of OSCC cells transfected with miR-146a-5p inhibitor compared to control cells at 24 and 48-hour time points determined by employing scratch assay. (**F**) Matrigel invasion assay showing a reduction in the invasive ability of OSCC cells transfected with miR-146a-5p inhibitor. Statistical comparisons of the data were made using the Student’s *t*-test and the data points represent the mean ± SEM. *P* < 0.05 was considered significant and the asterisk sign (^*^) denotes significant change compared to respective control samples.

miRNAs play pivotal roles in cancer pathophysiology, influencing processes such as cell proliferation, apoptosis, invasion, migration, angiogenesis, etc., [37]. Conflicting functional roles for miR-146a-5p in OSCC have been reported [28, 38]. We sought to delineate its pathophysiological function in OSCC, and to address this we resorted to a loss-of-function approach wherein miR-146a-5p was silenced in SCC-25 and SCC-9 cell lines using miRNA inhibitor. The knockdown of miR-146a-5p was validated by RT-qPCR, wherein >2.5-fold reduction in miR-146a-5p levels was observed in both the cell lines (Figure 3A). Upon miR-146a-5p depletion, the viability of SCC-25 and SCC-9 cells declined in a time-dependent manner, with ~16–22% reduction at 72 hours and ~29–32% reduction at 96 hours (Figure 3B). Further, significant activation of caspase 3/7 was observed in OSCC cells upon miR-146a-5p depletion, indicating enhanced apoptosis (Figure 3C). Akin to the proliferation data, the colony formation ability of SCC-25 and SCC-9 was also reduced upon miR-146a-5p depletion (Figure 3D). miR-146a-5p also impacts OSCC cell migration and invasion, key determinants of malignant progression and metastasis. The wound-healing assay revealed reduced migration in OSCC cells, while the invasion assay demonstrated a >30% reduction in the invasive ability of SCC-25 and SCC-9 cells when miR-146a-5p was inhibited (Figure 3E, 3F and Supplementary Figure 3A, 3B). In conclusion, these results unambiguously point to an oncogenic role of miR-146a-5p in OSCC cell lines by modulating proliferation, apoptosis, migration, and invasion.

### miR-146a-5p promotes OSCC progression by directly targeting SMAD4 3′UTR

**Figure 4:**
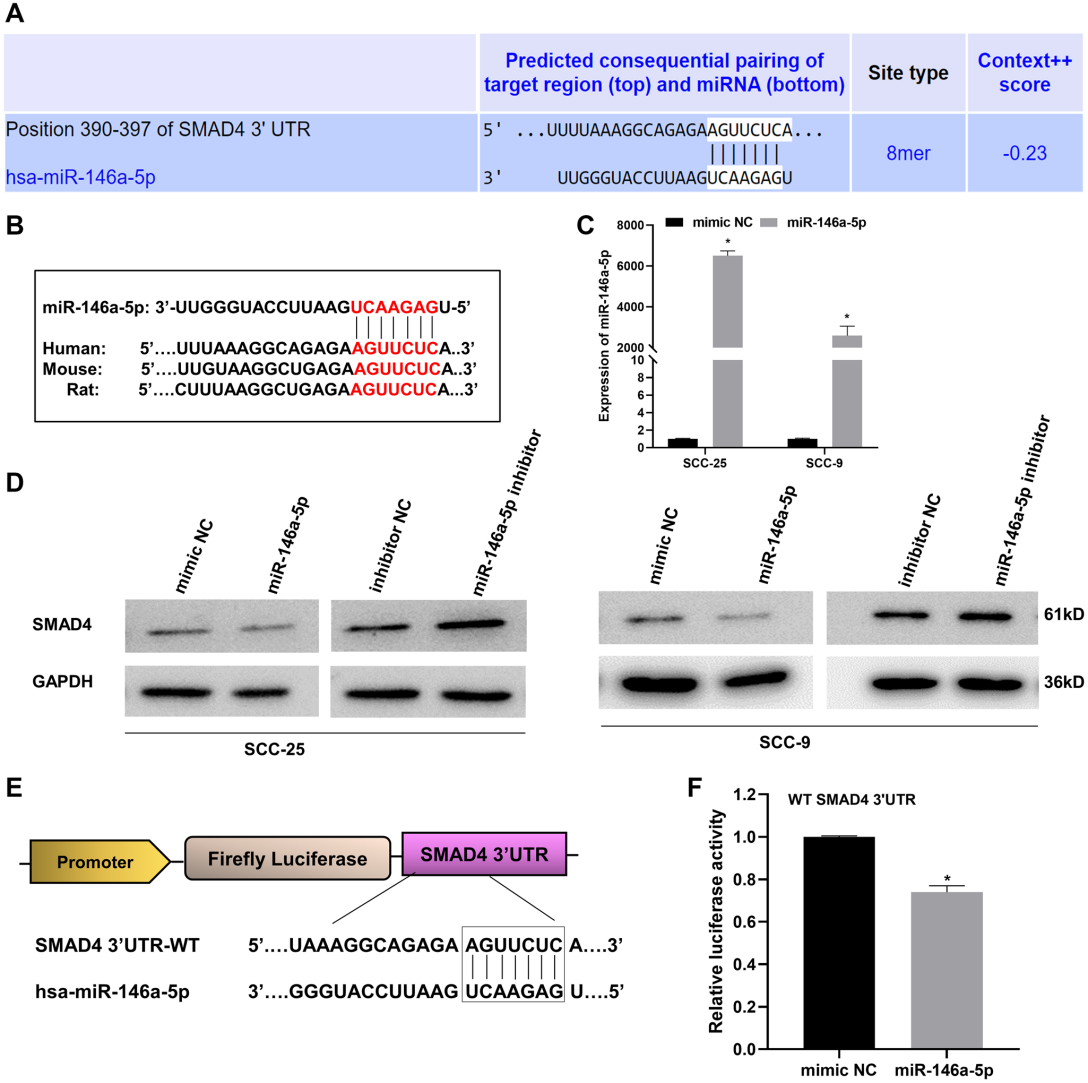
SMAD4 is a bona fide downstream target of miR-146a-5p. (**A**) The potential binding site of miR-146a-5p in the 3′UTR of SMAD4 as predicted by TargetScan. (**B**) The evolutionary conservation of the predicted miR-146a-5p binding sequence 5′-AGUUCUC-3′ across human, mouse, and rat SMAD4 3′UTRs. (**C**) Validation of miR-146a-5p overexpression in OSCC cell lines SCC-25 and SCC-9 by RT-qPCR. (**D**) Western blot assay showing the expression of SMAD4 protein in SCC-25 and SCC-9 cells upon inhibition and overexpression of miR-146a-5p by miRNA inhibitor and miRNA mimic respectively. (**E**) Schematic representation of the pmirGLO dual-luciferase reporter construct generated by cloning the SMAD4 3′UTR region harboring the predicted miR-146a-5p binding site downstream of the firefly luciferase gene. (**F**) Dual-luciferase reporter assay to validate the direct interaction between miR-146a-5p and SMAD4 3′UTR, carried out in HEK293T cells. The activity of firefly luciferase was normalized to the internal control, renilla luciferase. Statistical comparisons of the luciferase assay data were made using the Student’s *t*-test and the data points represent the mean ± SEM. *P* < 0.05 was considered significant and the asterisk sign (^*^) denotes significant change compared to the control sample.

miRNAs elicit their biological functions by base-pairing with the complementary sequences in the 3’UTR of target mRNAs to repress its expression at a post-transcriptional level [39]. To uncover the potential targets of miR-146a-5p, we conducted an exhaustive bioinformatics analysis, employing tools such as TargetScan, miRWalk, miRMap, and TarBase. One of the predicted targets of miR-146a-5p was SMAD4 mRNA, wherein miR-146a-5p binds to nucleotides 390-397 in the SMAD4 3′UTR with an 8-mer seed match (Figure 4A). Also, the binding sequence, 5′-AGUUCUC-3′, within SMAD4 3′UTR is evolutionarily conserved across humans, mice, and rats (Figure 4B). To probe the inverse correlation between miR-146a-5p and SMAD4, we examined the levels of SMAD4 protein following miR-146a-5p inhibition and overexpression in OSCC cells. miR-146a-5p overexpression upon transfecting its mimic was confirmed by RT-qPCR in SCC-25 and SCC-9 OSCC cells (Figure 4C). Overexpression of miR-146a-5p reduced the SMAD4 protein, whereas its inhibition increased SMAD4 protein levels in SCC-25 and SCC-9 cells (Figure 4D and Supplementary Figure 4). We further performed a dual-luciferase reporter assay to validate the direct binding relationship between miR-146a-5p and SMAD4. Toward this, we cloned the SMAD4 3′UTR region containing the predicted miR-146a-5p binding site into the pmirGLO dual-luciferase miRNA target expression vector downstream of the luciferase gene (Figure 4E). As expected, we observed a significant reduction (~26%) in the luciferase activity upon co-transfection of pmirGLO-SMAD4-3′UTR reporter plasmid and miR-146a-5p mimic (Figure 4F), confirming that SMAD4 is a bona fide downstream target of miR-146a-5p. Taken together, these results demonstrate that miR-146a-5p, a METTL3-regulated miRNA, directly and negatively regulates SMAD4 in OSCC cells by targeting its 3′UTR.

### SMAD4 silencing promotes OSCC progression

**Figure 5:**
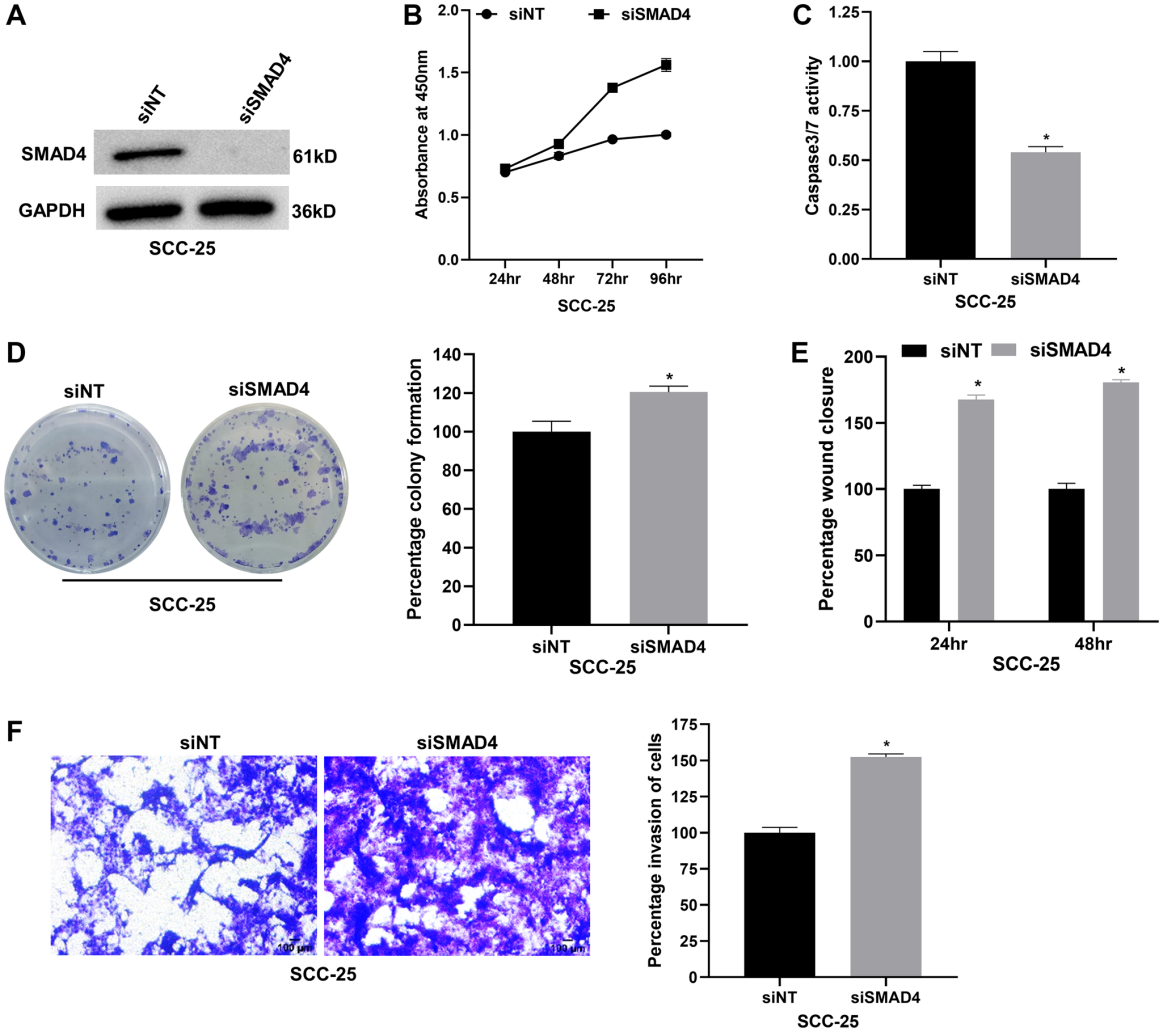
SMAD4 silencing promotes proliferation, migration, invasion and reduces apoptosis of the SCC-25 OSCC cell line *in vitro*. (**A**) Validation of siRNA-mediated knockdown of SMAD4 in SCC-25 cells by western blot assay. (**B**) WST-1 assay showing the effect of SMAD4 depletion in the cell viability of SCC-25 cells at various time points. (**C**) Probing apoptosis in SMAD4-depleted SCC-25 cells by caspase 3/7 luminometric assay. (**D**) Colony formation assay performed in SMAD4-depleted SCC-25 cells. (**E**) Percentage wound closure of SCC-25 cells transfected with siSMAD4 evaluated using scratch assay. (**F**) Matrigel invasion assay to examine the effect of SMAD4 knockdown in SCC-25 cell invasion. Statistical comparisons were made using the Student’s *t*-test and the data points represent the mean ± SEM. *P* < 0.05 was considered significant and the asterisk sign (^*^) denotes significant change compared to the control sample.

SMAD4, a well-known tumor suppressor gene frequently lost in many cancers, including OSCC, plays a pivotal role in several key signaling pathways, such as the TGF-β, BMP, and WNT pathways [40]. To discern the biological functions of SMAD4 in OSCC, we knocked down SMAD4 in SCC-25 and SCC-9 cells using siRNAs (Figure 5A and Supplementary Figure 5A) and performed various pathophysiological assays. We observe a time-dependent increase in cell viability upon SMAD4 depletion, with SCC-25 cells showing ~42% and ~55% increase at 72 and 96 hours, respectively (Figure 5B). Similarly, the cell viability of SCC-9 cells was observed to be increased by ~34% at 72 hours and ~36% at 96 hours (Supplementary Figure 5B). Further, SMAD4 inhibition reduced the activity of caspase 3/7 by 2-fold in SCC-25 cells, indicating a decrease in apoptosis (Figure 5C). Consistent with these findings, SMAD4 silencing enhanced the colony formation efficiency of SCC-25 cells (Figure 5D). Furthermore, SMAD4 suppression significantly impacted cell migration and invasion, wherein the wound-closing ability of SCC-25 and SCC-9 cells was increased by 80-85% at 48 hours (Figure 5E and Supplementary Figure 5C), and the invasion of SCC-25 and SCC-9 cells was enhanced upon depletion of SMAD4 (Figure 5F and Supplementary Figure 5D). Collectively, these results underscore the tumor-suppressive role of SMAD4 in OSCC cells by regulating cell proliferation, apoptosis, migration, and invasion.

### SMAD4 overexpression attenuates OSCC progression and its downstream targets are regulated by METTL3

**Figure 6:**
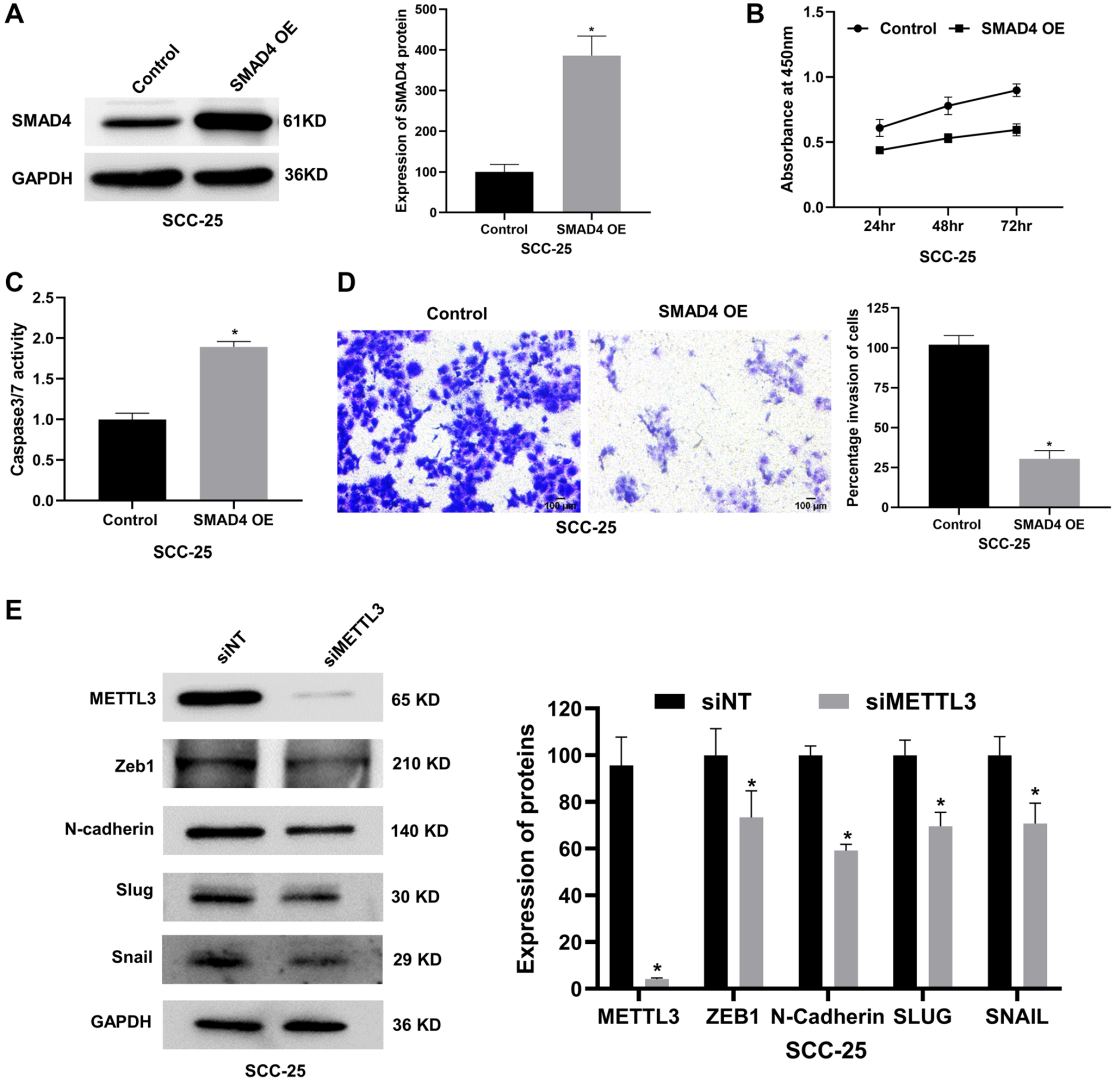
SMAD4 overexpression attenuates proliferation and invasion and elevates apoptosis of the SCC-25 cell line *in vitro* and METTL3-depletion regulates downstream effectors of SMAD4. (**A**) Validation of SMAD4 overexpression in SCC-25 cells by western blot assay. (**B**) WST-1 assay showing the effect of SMAD4 overexpression on the cell viability of SCC-25 cells at various time points. (**C**) Probing apoptosis in SMAD4-overexpressed SCC-25 cells by caspase 3/7 luminometric assay. (**D**) Matrigel invasion assay to examine the effect of SMAD4 overexpression in SCC-25 cell invasion. (**E**) Western blot assay showing the expression of downstream effectors of SMAD4-dependent TGF-β signaling such as SLUG, SNAIL, ZEB1, and N-cadherin in METTL3-depleted SCC-25 cells, along with corresponding quantification. Statistical comparisons were made using the Student’s *t*-test and the data points represent the mean ± SEM. *P* < 0.05 was considered significant and the asterisk sign (^*^) denotes significant change compared to the control sample.

Overexpressing SMAD4, a tumor suppressor gene is expected to reverse the cancerous phenotype exhibited by OSCC cells. Hence, we overexpressed SMAD4 in SCC-25 and SCC-9 OSCC cells employing a plasmid bearing SMAD4 cDNA. Western blot analysis confirmed a ~4-fold increase in SMAD4 protein levels in both cell lines (Figure 6A and Supplementary Figure 6A). SMAD4 overexpression led to a time-dependent reduction in cell viability, with SCC-25 cells showing ~30% and ~35% decrease at 48 and 72 hours, respectively (Figure 6B). A similar time-dependent reduction in cell viability was observed in SCC-9 cells upon SMAD4 overexpression (Supplementary Figure 6B). Further, SMAD4 overexpression increased the activity of caspase 3/7 by ~1.9-fold and ~1.4-fold in SCC-25 and SCC-9 cells, indicating an increase in apoptosis (Figure 6C and Supplementary Figure 6C), and significantly suppressed cell invasion by ~70% in SCC-25 and ~65% in SCC-9, suggestive of its potent role in suppressing OSCC cell invasion (Figure 6D and Supplementary Figure 6D). Collectively, these results underscore the tumor-suppressive role of SMAD4 in OSCC cells.

Since METTL3 is known to regulate TGF-β signaling in lung and gastric cancers [41, 42], and SMAD4 is a critical mediator of TGF-β signaling, we checked the expression levels of a few downstream effectors of SMAD4-dependent TGF-β signaling in METTL3-depleted SCC-25 cells, by western blot analysis. Interestingly, key transcription factors like SLUG, SNAIL, and ZEB1 and mesenchymal marker N-cadherin were observed to be downregulated upon METTL3-depletion in SCC-25 OSCC cells (Figure 6E), suggesting the involvement of METTL3 in SMAD4-dependent TGF-β signaling.

### METTL3/miR-146a-5p/SMAD4 axis regulates the progression of OSCC

**Figure 7:**
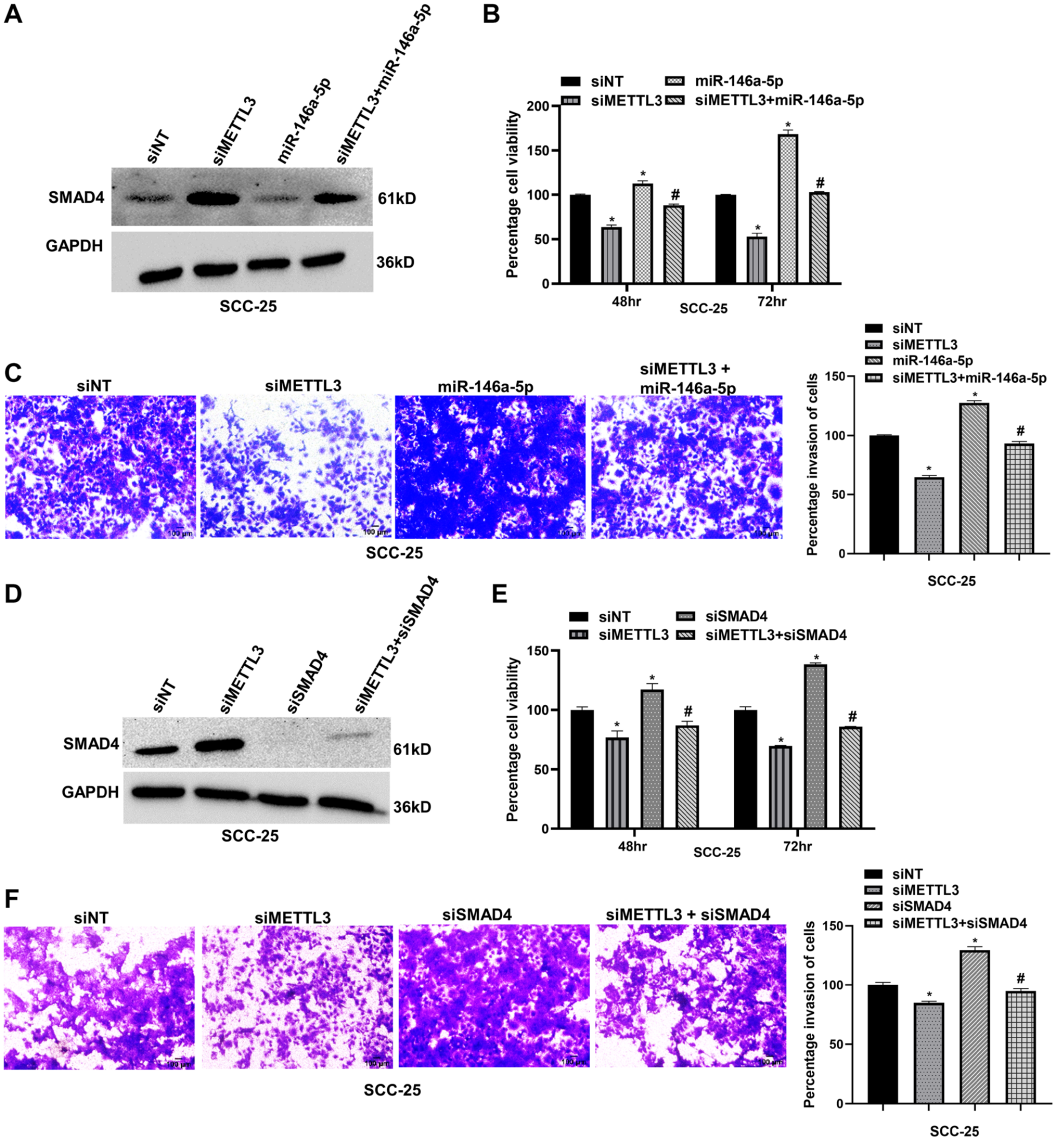
METTL3/miR-146a-5p/SMAD4 axis regulates OSCC progression. (**A**) Western blot analysis showing the impact of miR-146a-5p overexpression on siMETTL3 transfection-induced SMAD4 expression in SCC-25 cells. The co-transfection of miR-146a-5p mimic and METTL3 siRNA could partly reduce the SMAD4 protein expression level, induced by METTL3 knockdown. Quantification of SMAD4 protein levels is provided in Supplementary Figure 7A. (**B**) WST-1 assay showing the reduction in the cell viability of SCC-25 cells, caused due to METTL3 depletion, is rescued upon miR-146a-5p overexpression. (**C**) Matrigel invasion assay showing the effect of miR-146a-5p overexpression on METTL3 knockdown driven reduction in cell invasion of SCC-25 cells. (**D**) Western blot showing the effect of SMAD4 knockdown on METTL3-depletion-induced SMAD4 expression in SCC-25 cells. The double knockdown of SMAD4 and METTL3 could partially reduce METTL3-depletion-induced SMAD4 protein levels. Quantification of SMAD4 protein is provided in Supplementary Figure 7B. (**E**) WST-1 assay showing the reduction in the cell viability of SCC-25 cells caused due to METTL3 depletion is rescued upon SMAD4 knockdown. (**F**) Matrigel invasion assay showing the effect of SMAD4 knockdown on METTL3-depletion driven reduction in cell invasion of SCC-25 cells. Statistical comparisons were made using the Student’s *t*-test and the data points represent the mean ± SEM. *P* < 0.05 was considered significant. The asterisk sign (^*^) denotes a significant change compared to the control sample and the hash (^#^) sign denotes a significant change compared to siMETTL3.

To interrogate the role of the METTL3/miR-146a-5p/SMAD4 axis in OSCC progression, we performed a series of rescue experiments. The first set involved co-transfecting METTL3 siRNA and miR-146a-5p mimic to ascertain whether METTL3 modulates OSCC cell behavior via miR-146a-5p/SMAD4 regulation. The experimental groups included control, siRNA-mediated METTL3 knockdown, mimic-mediated miR-146a-5p overexpression, and co-transfection with siMETTL3 and miR-146a-5p mimic. Western blot analysis revealed that METTL3 knockdown elevated the expression of SMAD4 proteins, whereas miR-146a-5p overexpression reduced the expression of SMAD4 (Figure 7A and Supplementary Figure 7A). Notably, co-transfection with siMETTL3 and miR-146a-5p mimic reduced SMAD4 protein levels by ~64%, which increased due to METTL3 depletion (Figure 7A and Supplementary Figure 7A). Next, we checked the effect of co-transfection on cell viability and cell invasion properties of SCC-25 cells, and our data suggested that the overexpression of miR-146a-5p counteracted the decrease in cell proliferation and invasion caused by METTL3 silencing, with cell proliferation being restored by ~32% and ~64% at 48 and 72 hours, respectively (Figure 7B). Similarly, the invasion assay also displayed that miR-146a-5p overexpression increased SCC-25 cell invasion by ~36%, which had been suppressed by METTL3 knockdown (Figure 7C). Taken together, these observations convincingly show that miR-146a-5p overexpression negates the inhibitory effect of METTL3 silencing on OSCC cells, suggesting that METTL3 promotes OSCC through miR-146a-5p regulation.

A second set of rescue experiments was carried out to examine whether the phenotype induced by METTL3 depletion could be rescued by SMAD4 depletion. The transfection groups in these experiments were control, siRNA-mediated METTL3 knockdown, siRNA-mediated SMAD4 knockdown, and co-transfection with METTL3 and SMAD4 siRNAs. Western blot analysis revealed that METTL3 knockdown elevated the expression of SMAD4 protein levels and co-transfection of METTL3 and SMAD4 siRNAs reduced SMAD4 protein levels (by ~153%) increased due to METTL3 silencing (Figure 7D and Supplementary Figure 7B). Next, we probed the effect of co-transfection on the viability and invasion of SCC-25 cells. Interestingly, in cells co-transfected with METTL3 and SMAD4 siRNAs, cell viability was rescued by ~13% and ~21% at 48 and 72 hours, respectively (Figure 7E). Invasion assay also indicates that SMAD4 silencing increased the SCC-25 cell invasion by ~11%, counteracting the reduction caused by METTL3 knockdown (Figure 7F). Hence the observations from these rescue experiments are suggestive of a pivotal role for the METTL3/miR-146a-5p/SMAD4 axis in OSCC progression.

## DISCUSSION

OSCC represents a highly aggressive malignancy characterized by high morbidity, profound local invasion, distant metastasis, multidrug resistance, poor clinical outcomes, and a high recurrence rate. The asymptomatic progression of OSCC significantly impedes early detection, contributing to its substantial mortality burden [1]. Reversible m6A RNA methylation, a key epitranscriptomic modification of RNA, is important in cancer initiation, progression, and prognosis, including OSCC, with m6A components as well as m6A-regulated RNAs emerging as attractive potential therapeutic targets [43]. OSCC tissues and cell lines show aberrant expression of m6A components and elevated global transcriptome-wide m6A levels [44]. METTL3, the catalytic subunit of the m6A methyltransferase complex and the primary m6A RNA methyltransferase is upregulated in OSCC and associated with OSCC progression, poor prognosis, and reduced overall survival, in an m6A-dependent manner [7–9, 11, 13]. Functional studies have demonstrated that silencing METTL3 markedly reduces cell proliferation, migration, and invasion of OSCC cells *in vitro* and reduces tumor volume, tumor weight, proliferation, and lymphatic metastasis *in vivo* [7–9, 13]. Consistent with these observations, our findings confirm that METTL3 is overexpressed in OSCC cells, and its siRNA-mediated knockdown significantly impairs OSCC cell proliferation, migration, and invasion, while promoting apoptosis *in vitro* as evidenced by various pathophysiological assays, thereby affirming its oncogenic role in OSCC. Additionally, METTL3 depletion reduced the levels of cell cycle markers CCNB1 and CCND1 in OSCC cells. As crucial cyclins, CCNB1 drives cells from G2 to M phase and initiates mitotic progression, whereas CCND1 modulates the G1 to S transition and overexpression of these genes is frequently observed in multiple malignancies including OSCC [45, 46]. Therefore, decreased levels of these cyclins upon METTL3-depletion clearly indicates the involvement of METTL3 in OSCC cell cycle progression. METTL3 exerts its oncogenic role by regulating RNA transcripts in an m6A-dependent manner, including oncogenic mRNAs such as BMI1, cMYC, SLC7A11, SALL4, [7–9, 11], and non-coding regulatory miRNAs such as miR-99a-5p, and miR-31-5p in OSCC [22, 23].

METTL3 deposits m6A marks on pri-miRNAs, thereby facilitating their recognition by DGCR8 and enhancing their subsequent processing into mature miRNAs [17]. Huang et al., in 2023 characterized miR-99a-5p and miR-31-5p as METTL3 regulated in OSCC. Both miR-99a-5p and miR-31-5p are overexpressed in OSCC and promote OSCC progression by targeting ZBTB7A and HIPK2, respectively, in an m6A-dependent fashion [22, 23]. Akin to these previous studies, our data provide evidence for the first time that METTL3 regulates the maturation of miR-146a-5p in OSCC, and this regulation requires the catalytic activity of METTL3. We found that miR-146a-5p levels are significantly high in OSCC cells, which is consistent with its enhanced levels in bladder cancer, gastric cancer, thyroid cancer, breast cancer, cervical cancer, and NSCLC [34, 47–51]. We hypothesized that akin to previous reports, METTL3 might be regulating the biogenesis of miR-146a-5p via depositing m6A marks on pri-miR-146a and thus facilitating its recognition by DGCR8. The presence of several high confidence potential m6A sites in pri-miR-146a as predicted computationally, further lends support to this hypothesis. To uncover the regulatory relationship between METTL3 and miR-146a-5p in OSCC, we examined the impact of loss-of-function or chemically inhibiting the catalytic activity of METTL3 on the expression/levels of mature miR-146a-5p and pri-miR-146a. As expected, our data suggest METTL3 to be a positive regulator of miR-146a-5p expression/maturation, wherein depleting METTL3 or blocking its catalytic activity leads to an appreciable decrease in mature miR-146a-5p levels together with a concomitant accumulation of pri-miR-146a. Since inhibiting the catalytic activity of METTL3 yields a similar effect on mature miR-146a-5p levels as its siRNA-mediated knockdown, we infer that the regulation of miR-146a-5p maturation is dependent on m6A deposition by METTL3. To rule out the possibility of this regulation due to aberrant levels of DROSHA and DGCR8, critical components of miRNA biogenesis machinery, due to m6A RNA methylation, we examined their expression upon METTL3 depletion but did not observe any significant change (our unpublished data, data not shown). Our study identifies an m6A/METTL3-dependent epitranscriptomic mechanism as a contributor to miR-146a-5p overexpression in OSCC progression. However, the exceptionally high expression of miR-146a-5p in SCC-9 OSCC cells suggests additional regulatory mechanisms. Transcription factors like SNAIL and NF-kB, previously linked to miR-146a-5p regulation in other cancers, or rs2910164 polymorphism in pre-miR-146a reported in OSCC patients or activation of TNFα and TGF-β, may contribute to the high expression of miR-146a-5p in SCC-9 OSCC cells [52–54]. Further studies are required to uncover the roles of transcription factors, promoter polymorphisms, and other molecular pathways in driving miR-146a-5p overexpression in OSCC beyond METTL3-mediated regulation.

Conflicting reports exist regarding the precise functional role of miR-146a-5p in the context of OSCC [28, 38]. Our data unambiguously indicate an oncogenic role for miR-146a-5p in OSCC, wherein inhibition of miR-146a-5p attenuated oncogenic properties, and its overexpression exacerbated these effects. miRNAs often have dual functions in cancers, wherein they act as oncogenic activators and tumor suppressors by suppressing the expression of their target gene [39]. SMAD4 has been reported as one of the downstream targets of miR-146a-5p in gastric cancer, glioma, and prostate cancer [47, 55, 56]. However, the regulation of SMAD4 by miR-146a-5p is yet to be interrogated in OSCC. Similarly, the mechanisms behind the dysregulation of SMAD4, a key driver of OSCC, remains relatively unexplored. Given the tissue-specific nature of miRNAs, it is crucial to validate whether miR-146a-5p directly targets SMAD4 in OSCC cells. We validated SMAD4 to be a bona fide target of miR-146a-5p in OSCC cells by dual luciferase reporter assay and western blot assay. SMAD4, a highly conserved transcription factor and a central signaling transducer of the TGF-β pathway, is a well-known tumor-suppressor gene in several cancers and it functions by regulating tumorigenesis and differentiation [25, 57–59]. Levels of SMAD4 have been reported to be reduced in OSCC tissue samples and cell lines [27]. Consistent with previous findings, our data confirm its tumor-suppressive role in OSCC, as SMAD4 silencing enhances proliferation, migration, and invasion while reducing apoptosis, whereas its overexpression gives opposite effects.

**Figure 8:**
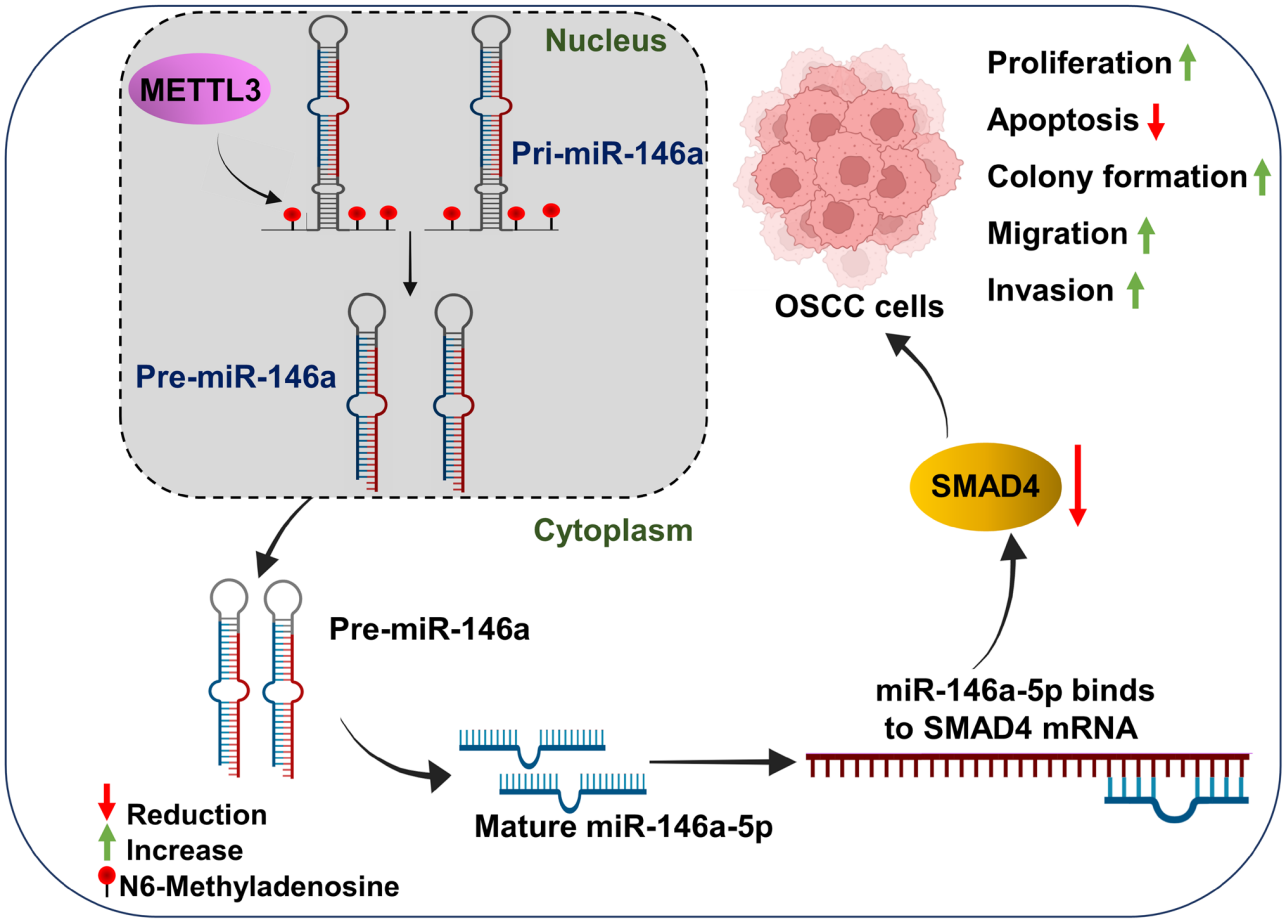
Schematic representation of METTL3-mediated OSCC progression via regulating the miR-146a-5p/SMAD4 axis. Overexpressed METTL3 (an oncogene) in OSCC cells enhances the processing of pri-miR-146a, leading to an increase in mature miR-146a-5p levels. This upregulated miR-146a-5p targets SMAD4 (a tumor-suppressor gene) via miRNA-mRNA binding interactions resulting in reduced SMAD4 protein expression. The reduction in the tumor-suppressor SMAD4 levels contributes to enhanced cell proliferation, colony formation, migration, invasion, and reduced apoptosis in OSCC cells.

SMAD4 inactivation through mutations or deletion makes it an attractive molecular marker in cancer pathogenesis [57]. SMAD4 has been reported to be frequently mutated in multiple oral cancer cell lines such as CAL-27, CAL-33, and UM-SCC-2, and is a driving factor for altering the antiproliferative function of TGF-β signaling in many cancers [60]. The expression of SMAD4 is reduced in 60% of OSCC cases, but interestingly SMAD4 mutation is reported only in 13% of the cases, suggestive of additional mechanisms such as miRNA-mediated regulation in modulating SMAD4’s expression [40]. However, to date, only a single miRNA, miR-558, has been identified to directly regulate SMAD4 expression in OSCC cells [57]. We report here a second SMAD4 targeting miRNA in OSCC i.e. miR-146a-5p, a METTL3 regulated miRNA to modulate the downregulation of SMAD4 in OSCC cells, wherein SMAD4 mutations are absent. Both the OSCC cell lines i.e., SCC-25 and SCC-9 used in our study bear wildtype SMAD4 locus [57, 61]. Taken together, in the present study we report a novel regulatory METTL3/miR-146a-5p/SMAD4 axis operative in OSCC cells, wherein METTL3 (an oncogene) regulates the expression of SMAD4 (a tumor-suppressor gene) via regulating the maturation of miR-146a-5p, in a m6A dependent manner (schematically depicted in Figure 8). Previous studies have implicated METTL3 and miR-146a-5p in the regulation of EMT markers across various cancers [62–64]. Further, METTL3 expression and m6A RNA methylation levels are enhanced during TGFβ-induced EMT in lung cancer [41]. Hence, we speculated that the METTL3/miR-146a-5p/SMAD4 regulatory axis could be critical for the EMT of OSCC cells via regulating TGF-β signaling. The TGF-β signaling pathway exhibits pleiotropic behavior, functioning as a tumor-suppressor in normal cells but transitioning to a tumor-promoting pathway under specific scenarios, one of which is the inactivation of SMAD4 [65]. Hence, we speculate that the METTL3/miR-146a-5p/SMAD4 axis could serve to facilitate this switch in OSCC, thereby converting the tumor-suppressive properties of TGF-β signaling into tumor-promotive capabilities. TGF-β promotes tumor progression by enhancing proliferation, remodeling the extracellular matrix, inducing aberrant EMT, facilitating metastasis, and enabling immune evasion [65]. To assess this, we analyzed the expression pattern of certain key downstream effectors of SMAD4-dependent TGF-β signaling and observed critical transcription factors such as SNAIL, SLUG and ZEB1, and a crucial EMT marker N-cadherin to be regulated by METTL3 in OSCC cells, wherein the levels of these downstream effectors were reduced upon METTL3-depletion in OSCC cells, suggesting the involvement of METTL3 in SMAD4-dependent TGF-β signaling.

Elevated levels of METTL3 correlate with poor prognosis and shorter survival of OSCC patients [8, 66]. miR-146a-5p expression was increased in 85% of OSCC tissue samples compared to normal tissues, with higher levels detected in pre-operative plasma than in post-operative plasma, suggesting that circulating miR-146a-5p originates from tumor tissues [35]. Notably, patients with lower miR-146a-5p expression exhibited better survival outcomes [35]. Further, SMAD4 loss is associated with poorer progression-free survival in OSCC patients [40]. Together these findings suggest that METTL3, miR-146a-5p, and SMAD4 hold potential as both diagnostic and prognostic biomarkers of OSCC.

METTL3 is a well-established oncogene in multiple cancers, which makes it an attractive therapeutic target. STM2457, a selective METTL3 inhibitor, has shown anti-tumor effects in many cancers including OSCC [16, 67–69]. Similarly, cancer therapies leveraging miRNA mimics to restore tumor-suppressive miRNAs or antimiRs to inhibit oncogenic miRNAs are under clinical investigation [70]. Therapeutic strategies such as inhibiting METTL3 with small-molecule inhibitors, targeting miR-146a-5p with an anti-miR-146a-5p approach, or restoring SMAD4 expression exogenously could potentially offer promising avenues for OSCC treatment. However, further studies to uncover additional coding/non-coding targets of METTL3, direct targets of miR-146a-5p, and upstream/downstream targets of SMAD4 responsible for its tumor-associated function in OSCC need to be probed.

## MATERIALS AND METHODS

### Cell lines and reagents

OSCC cell lines SCC-25 (ATCC-CRL-1628), SCC-9 (ATCC-CRL-1629), and the normal human oesophageal epithelial cell line Het-1A (ATCC-CRL-2692) were obtained from ATCC. All cell lines were cultured in humidified incubators at 37°C in the presence of 5% CO_2_. OSCC cell lines were propagated in Dulbecco’s Modified Eagle Medium: Nutrient Mixture F12 (DMEM/F-12; Thermo Fisher Scientific/GIBCO) supplemented with 10% Fetal Bovine Serum (FBS; Thermo Fisher Scientific/GIBCO), 100 IU/ml penicillin (Thermo Fisher Scientific/GIBCO) and 100 μg/ml streptomycin (Thermo Fisher Scientific/GIBCO). For SCC-9, the media was further supplemented with 400 ng/ml hydrocortisone (Sigma). Het-1A cells were cultured on plates coated with a mixture of 30 μg/ml collagen (Sigma), 10 μg/ml fibronectin (Sigma), and 10 μg/ml bovine serum albumin (Sigma) employing Bronchial Epithelial Cell Growth Medium BulletKit (Lonza, #CC-3170) supplemented with 100 IU/ml penicillin (Thermo Fisher Scientific/GIBCO) and 100 μg/ml streptomycin (Thermo Fisher Scientific/GIBCO). STM2457 (MedChemExpress, #HY-134836), an inhibitor of METTL3, was used at a concentration of 5 μM to treat OSCC cells to inhibit the catalytic activity of METTL3.

### siRNA and plasmid transfections

siRNAs were transfected using Lipofectamine RNAiMAX transfection reagent (Thermo Fisher Scientific/Invitrogen) as recommended by the manufacturer. Briefly, OSCC cells were plated in medium without antibiotics at appropriate cell density, and twelve hours post-plating, cells were transfected with 50 nM METTL3- or SMAD4-specific or control non-targeting siRNAs (Sigma-Aldrich, #VC30002N), and analyzed 72 hours post-transfection. The siRNA duplexes used in this study are listed in Supplementary Table 1. To overexpress or inhibit miR-146a-5p, we transfected OSCC cells with mimic of human miR-146a-5p (75 nM) (miRCURY LNA miRNA mimic, Qiagen, #339173) and with inhibitor of miR-146a-5p (75 nM) (miRCURY LNA miRNA inhibitor, Qiagen, #339121) using Lipofectamine RNAiMAX transfection reagent as per manufacturer’s instructions. miRCURY LNA miRNA mimic negative control (Qiagen, #339173) and miRCURY LNA miRNA inhibitor negative control (Qiagen, #339126) served as controls. Plasmids were transfected using jetPRIME transfection reagent (Polyplus/Sartorius) as recommended by the manufacturer. Briefly, OSCC cells were plated in medium without antibiotics at appropriate cell density, and twelve hours post-plating, cells were transfected with SMAD4 overexpression or control plasmids, transfection media changed after 6 hours and the cells were analyzed at the desired time point post-transfection.

### Plasmids

To construct the SMAD4 overexpression plasmid, human SMAD4 cDNA (NM_005359) was amplified by PCR using primers SMAD4_cDNA_EcoRI_FP/SMAD4_cDNA_XhoI_RP (Supplementary Table 1) and cloned into *EcoR*I-*Xho*I restricted pcDNA3.1(+) mammalian expression vector (Thermo Fisher Scientific). To generate SMAD4 3′UTR-luciferase reporter plasmid, the 3′UTR region of human SMAD4 containing the predicted miR-146a-5p binding site was PCR amplified using primers SMAD4_3UTR_SacI_FP/SMAD4_3UTR_XhoI_RP (Supplementary Table 1) and cloned into *Sac*I-*Xho*I restricted pmirGLO dual-luciferase miRNA target expression vector (Promega).

### RNA Isolation and RT-qPCR

Total RNA was isolated from cultured cells using RNAiso Plus (Takara, #9109) as recommended, treated with 50 U/ml of RQ1 DNase (Promega) at 37°C for 30 minutes to remove traces of genomic DNA and purified using RNA Clean and Concentrator-25 (Zymo Research, #R1018). First-strand cDNA synthesis (RT) was typically performed with 1 μg total RNA using the PrimeScript first-strand cDNA synthesis kit (Takara, #6110A) as per the manufacturer’s instructions. Quantitative PCR (qPCR) assays were done in triplicate using TB Green Premix Ex Taq II (Takara, #RR820A) and a LightCycler 480 PCR system (Roche) and the signals were normalized to GAPDH/TBP mRNA levels. The relative gene expression of each sample was calculated using the 2^–ΔΔCT^ formula. To quantitate miRNA expression, 2–5 μg total RNA was reverse transcribed to cDNA employing Mir-X miRNA First Strand Synthesis Kit (Takara, #638313) as recommended. miRNA qPCR assays were done in triplicate using a miRNA-specific 5’ forward primer and a universal mRQ 3’ reverse primer, and the signals were normalized to RNU44/SNORD25 snoRNA levels. All primer sequences are provided in Supplementary Table 1.

### Immunoblotting

OSCC cells were washed with ice-cold PBS and the proteins were extracted with 1X RIPA buffer (Millipore, #20-188) containing 1X protease inhibitor cocktail (Sigma, #S8820) and 1 mM PMSF (Sigma, #P7626). Cell lysates were incubated on ice for 20–30 min, with intermittent vortexing, and cleared by centrifugation at 20000 g at 4°C for 20 minutes. Protein concentration was determined using a BCA Protein Assay Kit (Thermo Scientific/Pierce). Equal amounts of protein samples were separated by 10% sodium dodecyl sulphate-polyacrylamide gel electrophoresis (SDS-PAGE), transferred to polyvinylidene fluoride (PVDF) membrane, followed by blocking with 5% skimmed milk (Millipore, #70166) at room temperature for 1 hour. The blots were incubated with the respective primary antibodies at 4°C overnight. The following primary antibodies were used: anti-METTL3 (1:3000, CST, #96391), anti-SMAD4 (1:1000, Santa Cruz, #sc-7966), anti-N-cadherin (1:2000, CST, #13116), anti-SNAIL (1:2500, CST, #C15D3), anti-SLUG (1:2500, Proteintech, #12129-1-AP), anti-ZEB1 (1:2000, Proteintech, #21544-1-AP), anti-GAPDH (1:20000, Proteintech, #60004-1-Ig). Blots were next incubated with secondary antibodies i.e., HRP-conjugated anti-rabbit (1:10000, Proteintech, #SA00001-2) or HRP-conjugated anti-mouse (1:10000, Proteintech, #SA00001-1) for 1 hour at room temperature, followed by visualization of the protein bands using Clarity Western ECL substrate (Bio-Rad, #170-5060), detection by VILBER Fusion Pulse ChemiDoc, and quantification by ImageJ software (National Institutes of Health, USA). GAPDH served as the housekeeping loading control for normalization.

### Cell proliferation assay

OSCC cells were seeded in 96-well plates at a concentration of 5000 cells/well, and transfected with siMETTL3 or siSMAD4 or miR-146a-5p mimic or inhibitor or SMAD4 overexpression plasmid, together with appropriate controls. Cell proliferation was measured at relevant time points by a colorimetric assay employing the WST-1 reagent (Roche, #05015944001) according to the manufacturer’s instructions. The absorbance was taken at 450 nm with a microplate reader to quantify cell proliferation.

### Colony formation assay

For colony formation assay, OSCC cells were seeded into 96-well plates and transfected with siMETTL3 or siSMAD4 or miR-146a-5p mimic or inhibitor, together with appropriate controls. 24 hours post-transfection, cells were trypsinized and seeded at a density of 500 cells per well and maintained at 37°C in a humidified incubator in the presence of 5% CO_2_ for 2 weeks. Media change was done every 3 days, and the colonies formed were fixed with methanol, followed by staining with crystal violet (Sigma, #V5265). The colonies were photographed and counted.

### Caspase 3/7 assay

OSCC cells were seeded in a 96-well plate and transfected with siMETTL3 or siSMAD4 or miR-146a-5p mimic or inhibitor or SMAD4 overexpression plasmid, together with appropriate controls. The enzymatic activity of caspase-3/7 in the transfected OSCC cells was measured using a luminometric assay kit for caspase-3/7 (Promega, #G8090) as recommended by the manufacturer. 48 hours post-transfection, pro-luciferin DEVD substrate and caspase-Glo 3/7 buffer were added to the cells, followed by a 30 minutes incubation and subsequent measurement of the luminescence using a SpectraMax iD3 microplate multimode reader (Molecular Devices).

### Wound healing assay

For wound healing assay, OSCC cells transfected with siMETTL3 or siSMAD4, or miR-146a-5p mimic or inhibitor, and appropriate controls were seeded in a 12-well plate and cultured overnight so that the cells formed a fully confluent monolayer. A scratch was made using a sterile 20 μl pipette tip in the centre of the wells, and the cell debris was washed off using PBS. Cells were maintained in 0.5% low serum-containing media and the scratch was imaged at 0-hour, 24-hour, and 48-hour time intervals. The percentage of wound closure i.e., the migrating length was measured using ImageJ (National Institutes of Health, USA).

### Invasion assay

For invasion assay, OSCC cells were seeded in 24-well plates and transfected with siMETTL3 or siSMAD4 or miR-146a-5p mimic or inhibitor or SMAD4 overexpression plasmid, together with appropriate controls. 24 hours post-transfection, cells were trypsinized and seeded to the Matrigel (Corning, #356234) coated upper chamber of transwell inserts (Corning, #3422) in serum-free media. Media containing 20% FBS was added to the lower chamber. 48 hours post-seeding, cells that remained in the upper chamber were removed by gentle swabbing and cells that invaded the lower chamber were fixed with 4% paraformaldehyde and stained with crystal violet (Sigma, #V5265). Images of stained invaded cells were captured using a Magnus INVI microscope and the percentage of invasion was calculated using ImageJ.

### Bioinformatics analysis

Potential downstream targets of miR-146a-5p were predicted by searching online TargetScan (https://www.targetscan.org/vert_80/), miRMap (https://mirmap.ezlab.org/) miRWalk (http://mirwalk.umm.uni-heidelberg.de/), and TarBase (http://diana.imis.athena-innovation.gr/DianaTools/index.php?r=tarbase/index) databases. Prediction of potential m6A sites was performed using SRAMP (http://www.cuilab.cn/sramp) online tool.

### Dual-luciferase assay

Direct interaction of miR-146a-5p with SMAD4 3’UTR was verified using the dual luciferase reporter assay. Briefly, HEK293T cells were seeded in a 48-well plate format at a density of 40000 cells/well, and 16 hours post-seeding cells were co-transfected with the pmirGLO-SMAD4-3′UTR luciferase reporter plasmid and miR-146a-5p mimic/mimic NC using Polyplus jetPRIME transfection reagent as per manufacturer’s protocol. 48 hours post-transfection, cells were lysed and the luciferase activity was measured using the Dual-Luciferase Reporter Assay System (Promega, #E1910) as recommended by the manufacturer. Luminescence was measured using the SpectraMax iD3 microplate multimode reader (Molecular Devices) and the data were normalized to Renilla luciferase, the internal control.

### Statistical analysis

GraphPad Prism program (v.8) was used to analyze and develop figures. The results are presented as mean ± SEM. For the comparison of the mean between 2 groups, the Student’s *t*-test has been used. *P*-value < 0.05 is considered statistically significant.

## SUPPLEMENTARY MATERIALS



## Abbreviations

OSCC: oral squamous cell carcinoma
m6A: N6-methyladenosine
METTL3: Methyltransferase-like 3
miRNA: microRNA
mRNA: messenger RNA
pri-miRNA: primary microRNA
EMT: epithelial-mesenchymal transition
DGCR8: DiGeorge syndrome critical region 8
TGFβ: transforming growth factor beta
BMP: bone morphogenetic protein
WNT: wingless-related integration site
SMAD4: mother’s against decapentaplegic homolog 4
CDK2: cyclin-dependent kinase 2
CCNB1: cyclin B1
CCND1: cyclin D1
SNAIL: snail family transcriptional repressor 1
SLUG: snail family transcriptional repressor 2
ZEB1: zinc finger E-box binding homeobox 1
ZEB2: zinc finger E-box binding homeobox 2
ATCC: American type culture collection
DMEM-F12: Dulbecco’s Modified Eagle Medium: Nutrient Mixture F12
PBS: phosphate buffered saline
FBS: fetal bovine serum
RT-qPCR: reverse transcriptase-quantitative PCR
siRNA: small interfering RNA
LNA: locked nucleic acid
UTR: untranslated region
RIPA: radioimmunoprecipitate assay buffer
PMSF: phenylmethylsulfonyl fluoride
BCA: bicinchoninic acid
SDS-PAGE: sodium dodecyl sulfate-polyacrylamide gel electrophoresis
PVDF: polyvinylidene fluoride
HRP: horseradish peroxidase
ECL: enhanced chemiluminescence
WST-1: water-soluble tetrazolium 1
SEM: standard error mean
nM: nanomolar
μM: micromolar
