# Supplementary material for: METTL3 promotes oral squamous cell carcinoma by regulating miR-146a-5p/SMAD4 axis
Source: Oncotarget. 2025 May 8;16:291–309. doi: 10.18632/oncotarget.28717 (PMC12060920; doi:10.18632/oncotarget.28717)
Supplement: Supplementary file 1 [file oncotarget-16-28717-s001.pdf]

METTL3 promotes oral squamous cell carcinoma by regulating miR-146a-5p/SMAD4 axis

SUPPLEMENTARY MATERIALS

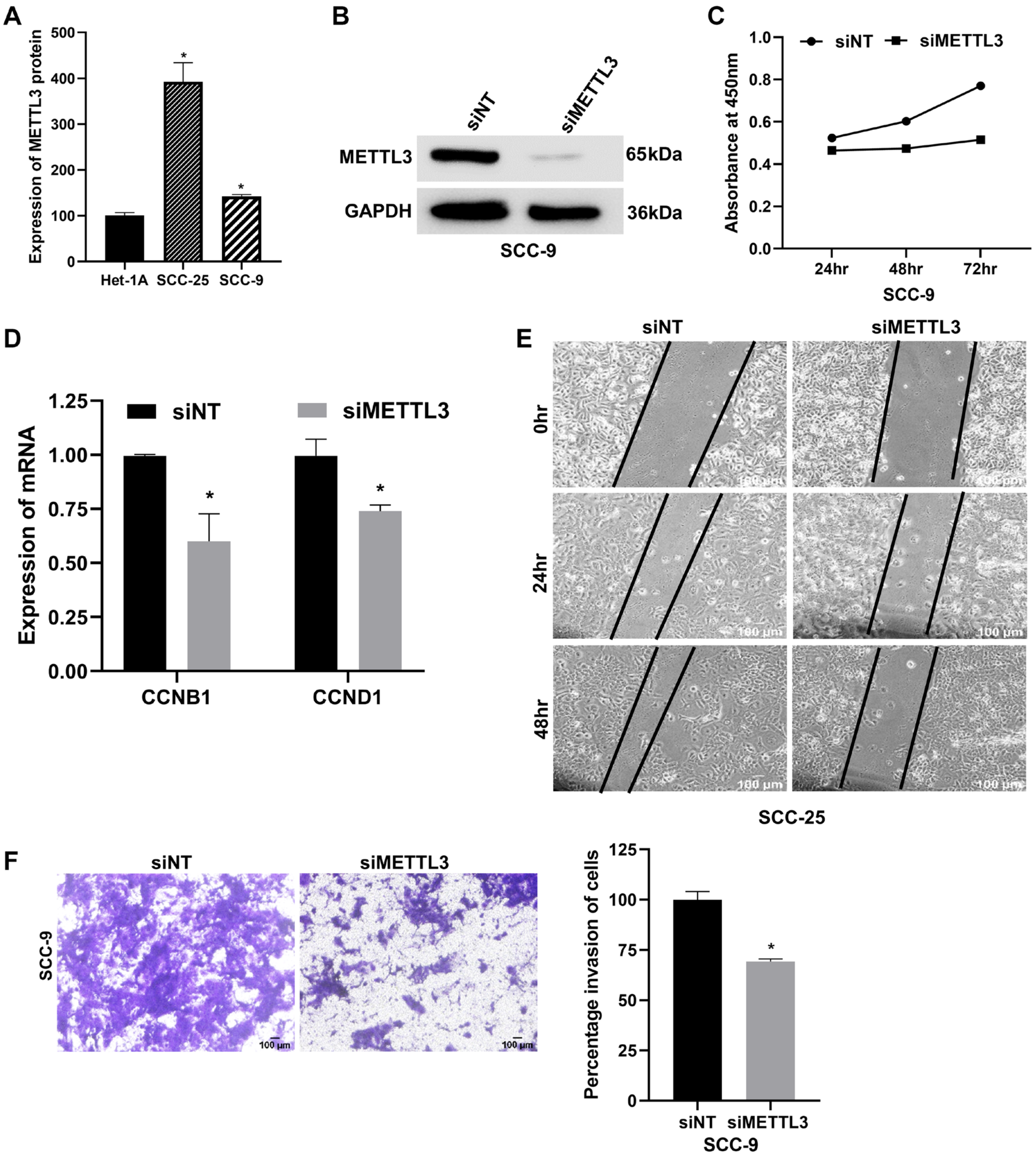

Supplementary Figure 1: Depletion of METTL3 regulates the progression of OSCC. (**A**) Quantification of western blot showing METTL3 protein levels in SCC-25 and SCC-9 cells compared to normal cell line Het-lA. (**B**) Validation of siRNA-mediated knockdown of METTL3 in SCC-9 cells by western blot assay. (**C**) WST-1 assay showing the effect of METTL3 depletion on cell viability of SCC-9 cells at various time points. (**D**) RT-qPCR showing the levels of cell cycle markers CCNBl and CCNDl in SCC-25 cells upon METTL3 depletion. (**E**) Representative images of wound healing assay performed in METTL3-depleted SCC-25 cells. The corresponding quantification of percentage wound closure done using ImageJ software is provided in Figure lF. (**F**) Matrigel invasion assay showing the effect of METTL3 knockdown in SCC-9 cell invasion, along with the corresponding quantification. Statistical comparisons were made using the Student's *t*-test and the data points represent the mean ± SEM. $P < 0.05$ was considered significant and the asterisk sign (*) denotes significant change compared to respective control samples.

A

#	Position	Sequence context	Structural context	Local structure visualization	Score (binary)	Score (knn)	Score (spectrum)	Score (combined)	Decision
1	82	UUUAU AACUC AUGAG UGCCA GGACU AGACC UGGUA CUAGG AAGCA	N/A	N/A	0.669	0.435	0.589	0.625	m6A site (High confidence)
2	165	UACAG GGCUG GGACA GGCCU GGACU GCAAG GAGGG GUCUU UGCAC	N/A	N/A	0.693	0.566	0.436	0.583	m6A site (Moderate confidence)
3	226	AUGUG UAUCC UCAGC UUUGA GAACU GAAUU CCAUG GGUUG UGUCA	N/A		0.606	0.443	0.46	0.539	m6A site (Low confidence)
4	335	UGGAG AGAGU AGAUC CUGAA GAACU UUUUC AGUCU GCUGA AGAGC	N/A	N/A	0.728	0.736	0.412	0.602	m6A site (High confidence)
5	428	GGAGU GUGAG UUCCU GUGAG AAACA CUCAU UUGAU UGUGA AAAGA	N/A	N/A	0.72	0.154	0.440	0.579	m6A site (Moderate confidence)
6	450	ACACU CAUUU GAUUG UGAAA AGACU UGAAU UCUAU GCUAA GCAGG	N/A	N/A	0.870	0.201	0.466	0.675	m6A site (Very high confidence)

B

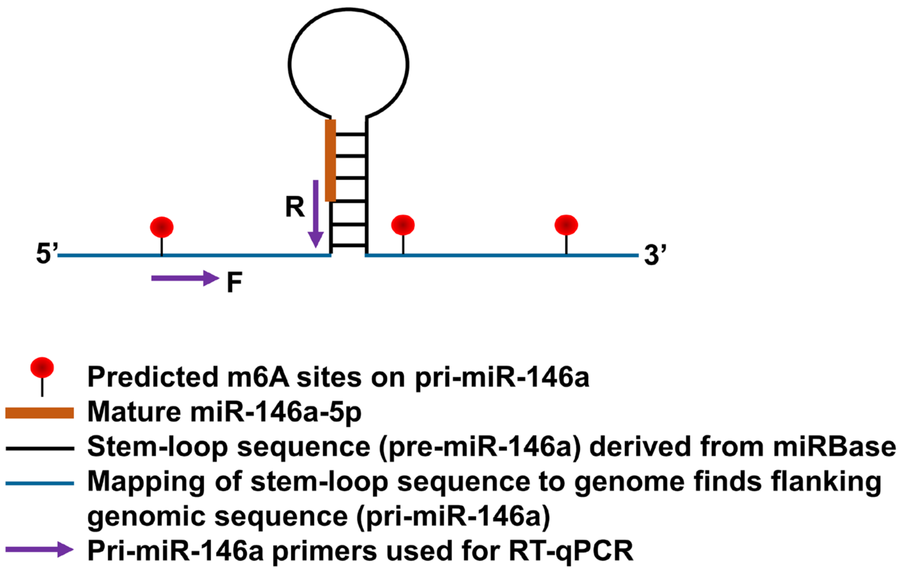

Supplementary Figure 2: m6A RNA methylation on pri-miR-146a. (**A**) The potential m6A motifs on pri-miR-146a predicted by the bioinformatics tool SRAMP. The 499 nucleotide long sequence of pri-miR-146a was obtained from UCSC with 200 nucleotides flanking upstream and downstream of the pre-miR-146a stem-loop for the prediction. A total of 6 m6A sites were predicted with three of them classified as high-confidence sites. (**B**) Schematic representation showing the location of the predicted high-confidence m6A sites on pri-miR-146a, along with the RT-qPCR primers used for detecting pri-miR-146a expression.

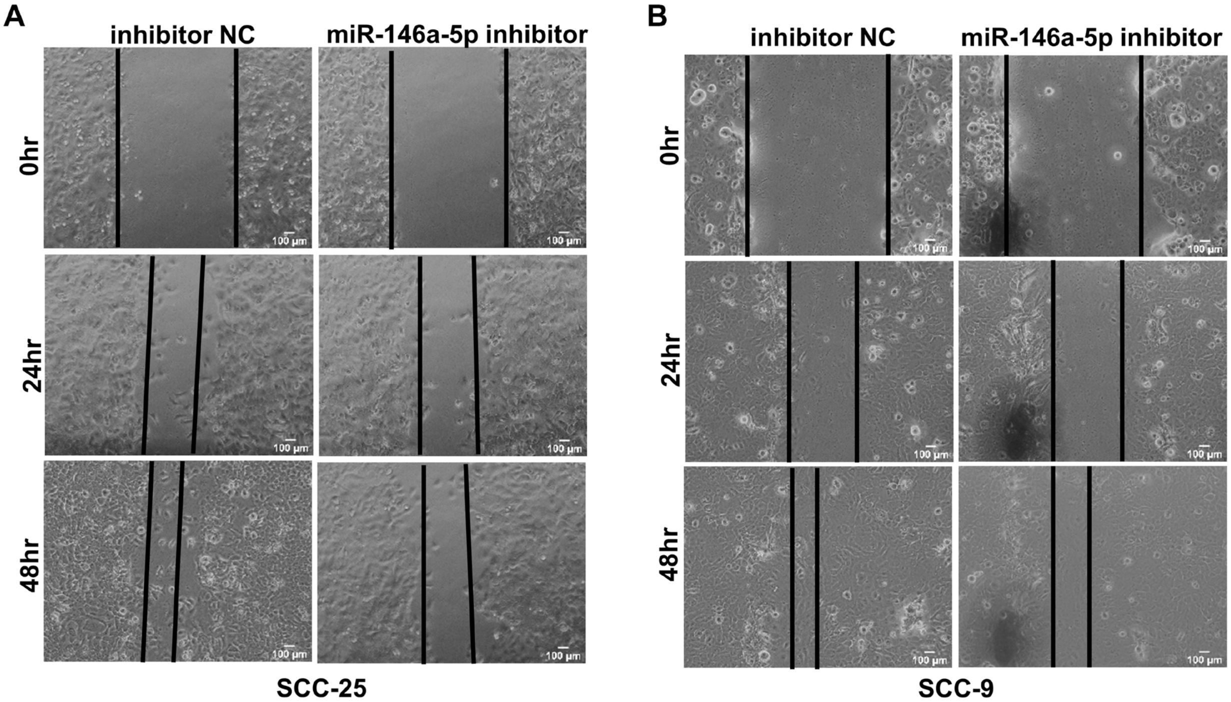

Supplementary Figure 3: miR-146a-5p inhibition regulates the progression of OSCC. (**A**) Representative images of wound healing assay performed in miR-146a-5p inhibited SCC-25 cells at the mentioned time points. (**B**) Representative images of wound healing assay performed in miR-146a-5p inhibited SCC-9 cells at the mentioned time points. The corresponding quantification is provided in Figure 3D.

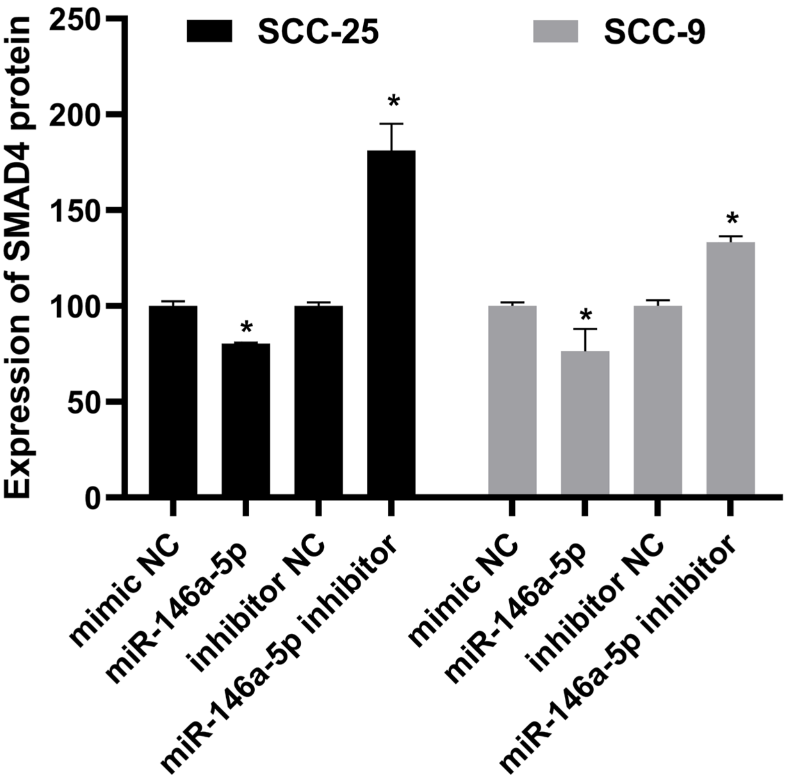

Supplementary Figure 4: Quantification of SMAD4 protein levels upon miR-146a-5p overexpression or inhibition in OSCC cells. The relative expression of SMAD4 protein is analyzed by normalizing to GAPDH expression, the internal control, using ImageJ software. Statistical comparisons were made using the Student's *t*-test and the data points represent the mean± SEM. $P < 0.05$ was considered significant and the asterisk sign (*) denotes significant change compared to respective control samples.

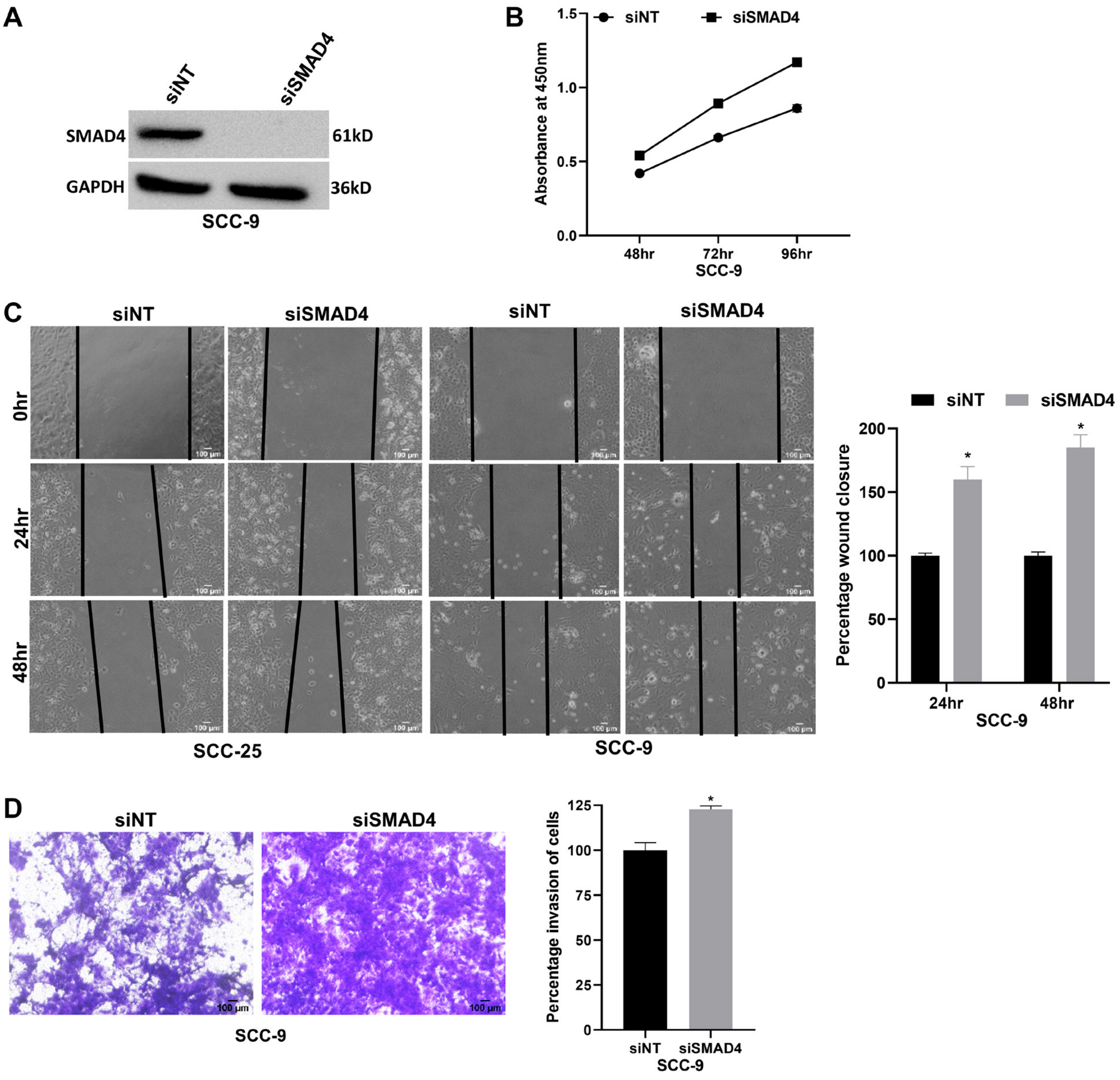

Supplementary Figure 5: Effect of SMAD4 knockdown on OSCC progression. (**A**) Validation of siRNA-mediated knockdown of SMAD4 in SCC-9 cells by western blot assay. (**B**) WST-1 assay showing the effect of SMAD4 depletion on cell viability of SCC-9 cells at different time points. (**C**) Representative images of wound healing assay performed in SMAD4-depleted SCC-25 and SCC-9 cells, along with the quantification of percentage cell migration in SCC-9 cells. The quantification of the percentage migration of SCC-25 cells upon SMAD4 depletion is provided in Figure 5E. (**D**) Matrigel invasion assay showing the effect of SMAD4 knockdown in SCC-9 cell invasion, along with the corresponding quantification. Statistical comparisons were made using the Student's *t*-test and the data points represent the mean ± SEM. $P < 0.05$ was considered significant and the asterisk sign (*) denotes significant change compared to respective control samples.

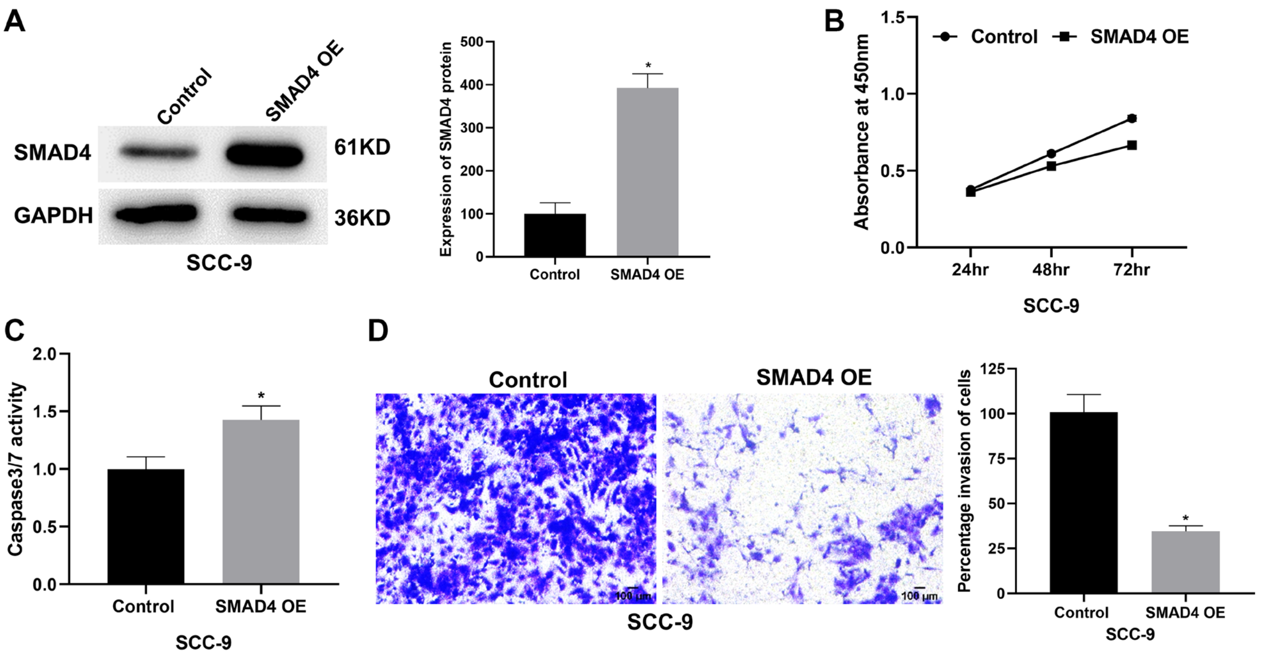

Supplementary Figure 6: Overexpression of SMAD4 regulates OSCC progression and METTL3 regulates downstream effectors of SMAD4. (**A**) Validation of SMAD4 overexpression in SCC-9 cells upon transfection of SMAD4 overexpression plasmid by western blot analysis. (**B**) WST-1 assay showing the effect of SMAD4 overexpression on cell viability of SCC-9 cells at different time points. (**C**) Caspase 3/7 assay showing effect of SMAD4 overexpression on SCC-9 cell apoptosis. (**D**) Matrigel invasion assay showing the effect of SMAD4 overexpression in SCC-9 cell invasion, along with the corresponding quantification. Statistical comparisons were made using the Student's *t*-test and the data points represent the mean ± SEM. $P < 0.05$ was considered significant and the asterisk sign (*) denotes significant change compared to respective control samples.

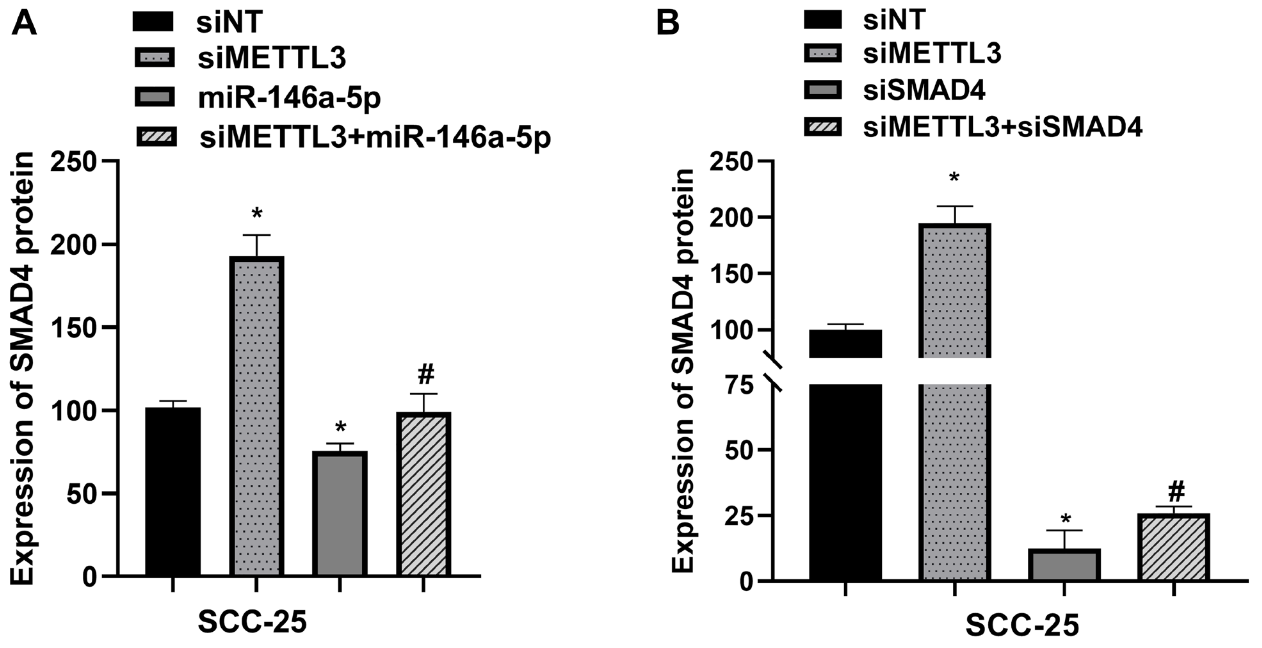

Supplementary Figure 7: Quantification of SMAD4 protein levels in SCC-25 cells following co-transfection with METTL3 siRNA and miR-146a-5p mimic or SMAD4 siRNA. (**A**) Quantification of western blot showing SMAD4 protein levels upon co-transfection of METTL3 siRNA and miR-146a-5p mimic. (**B**) Quantification of western blot analysis showing SMAD4 protein levels upon co-transfection of METTL3 and SMAD4 siRNAs. SMAD4 expression was normalized to GAPDH as the internal control and calculations were made using ImageJ software. Statistical comparisons were made using the Student's *t*-test and the data points represent the mean ± SEM. $P < 0.05$ was considered significant. The asterisk sign (*) denotes a significant change compared to the control sample and the hash (#) sign denotes a significant change compared to siMETTL3.

Supplementary Table 1: siRNAs and DNA oligonucleotides used in this study

Name	Sequence (5′ to 3′)
**siRNAs for METTL3 and SMAD4 knockdown**	
siMETTL3 sense	CUGCAAGUAUGUUCACUAUGA
siMETTL3 antisense	UCAUAGUGAACAUACUUGCAG
siSMAD4 sense	GUGUGCAGUUGGAAUGUAA
siSMAD4 antisense	UUACAUUCCAACUGCACAC
siNT sense	UAGCGACUAAACACAUCAA
siNT antisense	UUGAUGUGUUUAGUCGCUA
**Primers used for RT-qPCR analyses**	
METTL3_FP	CTCTATCCAGGCCCACAAGAAG
METTL3_RP	GTCACTACGGAAGGTTGGAGAC
SMAD4_FP	AAGGTGATGTTTGGGTCAGGTG
SMAD4_RP	TCGATGACACTGACGCAAATCA
CCNB1_FP	GGCCTCTACCTTTGCACTTCC
CCNB1_RP	GGAGGAAAGTGCACCATGTCA
CCND1_FP	GGAGCCCCAACAACTTCCTG
CCND1_RP	CTCCTCTTCCTCCTCCTCGG
GAPDH_FP	CGCTCTCTGCTCCTCCTGTT
GAPDH_RP	CCATGGTGTCTGAGCGATGT
TBP_FP	GGTTTTCCAGCTAAGTTCTTGGA
TBP_RP	AAGGAGAACAATTCTGGGTTTGA
pri-miR-146a_FP	CAGGACTAGACCTGGTACTAGGAAG
pri-miR-146a_RP	GTTCTCAAAGCTGAGGATACACATC
miR-146a-5p_FP	TGAGAACTGAATTCCATGGGTTA
RNU44_FP	GCAAATGCTGACTGAACATGAA
SNORD25_FP	TGTACTGAGCTCCGTGAGGA
mRQ 3′_RP	GCAGTGGTATCAACGCAGAGTAC
**Primers used for cloning SMAD4 3′UTR and SMAD4 cDNA**	
SMAD4_3UTR_SacI_FP	CGAGAGCTCTTGGGGCCCTTAACCTTATCAG
SMAD4_3UTR_XhoI_RP	CCGCTCGAGAAGGGATTCTCAATATACACACAGA
SMAD4_cDNA_EcoRI_FP	CGGAATTCATGGACAATATGTCTATTACGAATACA
SMAD4_cDNA_XhoI_RP	CCGCTCGAGTCAGTCTAAAGGTTGTGGGTCTGCAATC
